# Neuronal and glial 3D chromatin architecture informs the cellular etiology of brain disorders

**DOI:** 10.1038/s41467-021-24243-0

**Published:** 2021-06-25

**Authors:** Benxia Hu, Hyejung Won, Won Mah, Royce B. Park, Bibi Kassim, Keeley Spiess, Alexey Kozlenkov, Cheynna A. Crowley, Sirisha Pochareddy, The PsychENCODE Consortium, Allison E. Ashley-Koch, Gregory E. Crawford, Melanie E. Garrett, Lingyun Song, Alexias Safi, Graham D. Johnson, Gregory A. Wray, Timothy E. Reddy, Fernando S. Goes, Peter Zandi, Julien Bryois, Andrew E. Jaffe, Amanda J. Price, Nikolay A. Ivanov, Leonardo Collado-Torres, Thomas M. Hyde, Emily E. Burke, Joel E. Kleiman, Ran Tao, Joo Heon Shin, Kiran Girdhar, Yan Jiang, Marija Kundakovic, Leanne Brown, Jennifer R. Wiseman, Elizabeth Zharovsky, Rivka Jacobov, Olivia Devillers, Elie Flatow, Gabriel E. Hoffman, Judson Belmont, Diane Del Valle, Nancy Francoeur, Evi Hadjimichael, Dalila Pinto, Harm van Bakel, Panos Roussos, John F. Fullard, Jaroslav Bendl, Mads E. Hauberg, Alexander W. Charney, Vahram Haroutunian, Barbara K. Lipska, David A. Lewis, Chang-Gyu Hahn, Lara M. Mangravite, Mette A. Peters, Yooree Chae, Junmin Peng, Mingming Niu, Xusheng Wang, Maree J. Webster, Thomas G. Beach, Chao Chen, Yi Jiang, Rujia Dai, Yongjun Wang, Yan Xia, Annie W. Shieh, Chunyu Liu, Kay S. Grennan, Ramu Vadukapuram, Gina Giase, Dominic Fitzgerald, Lijun Cheng, Miguel Brown, Mimi Brown, Tonya Brunetti, Thomas Goodman, Majd Alsayed, Kevin P. White, Mohana Ray, Damon Polioudakis, Brie Wamsley, Jiani Yin, Luis De La Torre Ubieta, Michael J. Gandal, Vivek Swarup, Stephan J. Sanders, Matthew W. State, Donna M. Werling, Joon-Yong An, Brooke Sheppard, A. Jeremy Willsey, Amira Kefi, Eugenio Mattei, Michael Purcaro, Zhiping Weng, Jill Moore, Henry Pratt, Jack Huey, Tyler Borrman, Patrick F. Sullivan, Paola Giusti-Rodriguez, Yunjung Kim, Jin Szatkiewicz, Suhn Kyong Rhie, Christoper Armoskus, Adrian Camarena, Peggy J. Farnham, Valeria N. Spitsyna, Heather Witt, Shannon Schreiner, Oleg V. Evgrafov, James A. Knowles, Mark Gerstein, Shuang Liu, Fabio C. P. Navarro, Jonathan Warrell, Declan Clarke, Prashant S. Emani, Mengting Gu, Xu Shi, Min Xu, Yucheng T. Yang, Robert R. Kitchen, Gamze Gürsoy, Jing Zhang, Becky C. Carlyle, Angus C. Nairn, Mingfeng Li, Mario Skarica, Zhen Li, Andre M. M. Sousa, Gabriel Santpere, Jinmyung Choi, Ying Zhu, Tianliuyun Gao, Daniel J. Miller, Adriana Cherskov, Mo Yang, Anahita Amiri, Gianfilippo Coppola, Jessica Mariani, Soraya Scuderi, Anna Szekely, Flora M. Vaccarino, Feinan Wu, Sherman Weissman, Daifeng Wang, Tanmoy Roychowdhury, Alexej Abyzov, Yun Li, Stella Dracheva, Nenad Sestan, Schahram Akbarian, Daniel H. Geschwind

**Affiliations:** 1 UNC Neuroscience Center, University of North Carolina, Chapel Hill, NC USA; 2 Department of Genetics, University of North Carolina, Chapel Hill, NC USA; 3 Friedman Brain Institute, Icahn School of Medicine at Mount Sinai, New York, NY USA; 4 Department of Psychiatry, Icahn School of Medicine at Mount Sinai, New York, NY USA; 5 James J. Peters VA Medical Center, Bronx, NY USA; 6 Department of Neuroscience and Kavli Institute for Neuroscience, Yale School of Medicine, New Haven, CT USA; 7 Biostatistics, University of North Carolina, Chapel Hill, NC USA; 8 Computer Science, University of North Carolina, Chapel Hill, NC USA; 9 Department of Psychiatry, Yale School of Medicine, New Haven, CT USA; 10 Department of Genetics, Yale School of Medicine, New Haven, CT USA; 11 Department of Comparative Medicine, Program in Integrative Cell Signaling and Neurobiology of Metabolism, Yale School of Medicine, New Haven, CT USA; 12 Program in Cellular Neuroscience, Neurodegeneration, and Repair and Yale Child Study Center, Yale School of Medicine, New Haven, CT USA; 13 Neurogenetics Program, Department of Neurology, David Geffen School of Medicine University of California, Los Angeles, CA USA; 14 Center for Autism Research and Treatment, Semel Institute, David Geffen School of Medicine University of California, Los Angeles, CA 90095 USA; 15 Department of Human Genetics, David Geffen School of Medicine University of California, Los Angeles, CA USA; 16 Department of Psychiatry and Biobehavioral Sciences, Semel Institute, David Geffen School of Medicine University of California, Los Angeles, CA USA; 17 Duke University, Durham, NC USA; 18 Johns Hopkins University, Baltimore, MD USA; 19 Karolinska Institutet, Stockholm, Sweden; 20 Lieber Institute for Brain Development, Baltimore, MD USA; 21 Human Brain Collection Core, National Institutes of Health, Bethesda, MD USA; 22 University of Pittsburgh, Pittsburg, PA USA; 23 University of Pennsylvania, Philadelphia, PA USA; 24 Sage Bionetworks, Seattle, WA USA; 25 St. Jude Children’s Hospital, Memphis, TN USA; 26 Stanley Medical Research Institute, Kensington, MD USA; 27 Banner Sun Health Research Institute, Sun City, AZ USA; 28 Central South University, Changsha, Hunan China; 29 SUNY Upstate Medical University, Syracuse, NY USA; 30 The University of Chicago, Chicago, IL USA; 31 University of California–San Francisco, San Francisco, CA USA; 32 University of Illinois at Chicago, Chicago, IL USA; 33 University of Massachusetts Medical School, Worcester, MA USA; 34 University of Southern California, Los Angeles, CA USA; 35 SUNY Downstate Medical Center, Brooklyn, NY USA; 36 Program in Computational Biology and Bioinformatics, Yale University, New Haven, CT USA; 37 University of Wisconsin-Madison, Madison, WI USA; 38 Mayo Clinic Rochester, Rochester, MN USA

**Keywords:** Epigenetics, Epigenomics, Chromatin, Epigenetics in the nervous system, Genetics of the nervous system

## Abstract

Cellular heterogeneity in the human brain obscures the identification of robust cellular regulatory networks, which is necessary to understand the function of non-coding elements and the impact of non-coding genetic variation. Here we integrate genome-wide chromosome conformation data from purified neurons and glia with transcriptomic and enhancer profiles, to characterize the gene regulatory landscape of two major cell classes in the human brain. We then leverage cell-type-specific regulatory landscapes to gain insight into the cellular etiology of several brain disorders. We find that Alzheimer’s disease (AD)-associated epigenetic dysregulation is linked to neurons and oligodendrocytes, whereas genetic risk factors for AD highlighted microglia, suggesting that different cell types may contribute to disease risk, via different mechanisms. Moreover, integration of glutamatergic and GABAergic regulatory maps with genetic risk factors for schizophrenia (SCZ) and bipolar disorder (BD) identifies shared (parvalbumin-expressing interneurons) and distinct cellular etiologies (upper layer neurons for BD, and deeper layer projection neurons for SCZ). Collectively, these findings shed new light on cell-type-specific gene regulatory networks in brain disorders.

## Abstract (web summary)

The cellular heterogeneity in brain obscures the identification of robust cellular regulatory networks. Here the authors integrate genome-wide chromosome conformation data from sorted neurons and glia, with transcriptomic and enhancer profiles, to characterize cell-type-specific gene regulatory landscapes in the human brain, and provide insights into cell-type-specific gene regulatory networks in brain disorders.

## Introduction

The majority of human genetic variants imparting risk for brain diseases are located within non-coding elements^1^. Allelic variation in these elements is thought to have an influence on complex human traits via impacting gene regulation^2^, necessitating the understanding of gene regulatory architecture in the human brain. We and others have identified gene regulatory relationships in the developing and adult human brain by integrating multi-dimensional datasets that include transcriptomic, epigenomic, and higher-order chromatin interaction landscapes^3–7^. However, cellular heterogeneity poses significant challenges for unraveling the complexity in the gene regulatory architecture of the human brain, as it is comprised of heterogeneous cell populations, mostly different types of neurons and glia, each of which display distinct gene expression^3,8–10^ and chromatin accessibility profiles^11–14^. To address this issue, several groups have employed Hi–C and its derivatives (e.g. promoter-capture Hi–C) to build higher-order chromatin interaction maps in induced pluripotent stem cells (iPSC)-derived neurons and astrocytes^15^. However, these published studies rely on in vitro cultured cells that mark early brain development. Recently, promoter interaction profiles were inferred from three types of brain cells (neurons, oligodendrocytes, and microglia) obtained from the postnatal human cerebral cortex^16^.

Here, we analyze genome-wide chromosome conformation at cellular resolution to capture how chromatin structure affects cellular expression profiles. We use fluorescence-activated nuclear sorting (FANS, “Methods”)^17^ to sort neurons (NeuN+ cells) and glia (NeuN− cells), two major cell classes in the brain, and generated genome-wide chromosome conformation using Hi–C (Supplementary Figs. 1–2). We call multiple architectural units: A/B compartments, which are mutually exclusive large-scale chromatin domains, where compartment A is associated with actively transcribed regions and compartment B is enriched with repressive chromatin regions; Topologically Associating Domains (TADs), which are enriched with self-contacts and thus delineate the genomic regions within which most *cis*-regulatory interactions occur; Frequently Interacting REgions (FIREs), which are local interaction hotspots that are enriched with active regulatory elements; and gene loops within NeuN+ and NeuN− cells which link promotors to presumed regulatory elements (Supplementary Fig. 1). Furthermore, we integrate acetylated histone H3 lysine 27 (H3K27ac) peaks from glutamatergic (Glu) and medial ganglionic eminence (MGE)-derived GABAergic (GABA) neurons^18^ with NeuN+ chromatin interactions to obtain finer-scale gene regulatory relationship within these two major neuronal subtypes. We then leveraged cell-type-specific gene regulatory relationships to help decipher the genetic mechanisms contributing to Alzheimer’s disease (AD), schizophrenia (SCZ), and bipolar disorder (BD). Our results illustrate deciphering the epigenetic landscape in a cell-type-specific fashion offers substantial advantages for inferring the functional impact of genetic risk factors associated with brain disorders.

## Results

### Differential FIREs and super-FIREs are associated with cell-type-specific gene regulation

Hi–C libraries were generated from NeuN+ and NeuN− cells sorted from four dorsolateral prefrontal cortex (DLPFC) samples (Supplementary Table 1 and Supplementary Fig. 2). We first measured the reproducibility across Hi–C libraries using a stratum-adjusted correlation coefficient (SCC) that systematically quantifies similarities between two Hi–C contact maps^19^. Notably, we found that samples were clustered by cell types, not individuals, and showed high correlation between the same cell types from different individuals (NeuN+ = 0.95–0.97; NeuN− = 0.86-0.91; Supplementary Fig. 3a), demonstrating the reproducibility across Hi–C libraries.

We also compared the chromatin contacts of NeuN+ and NeuN− cells with other existing Hi–C datasets from brain-relevant tissues (Supplementary Fig. 3b, “Methods”). We found that NeuN− cells showed higher structural similarity with the adult brain than the fetal brain, indicative of gliogenesis in the postnatal brain^20^. Intriguingly, NeuN− cells did not show high structural similarity with iPSC-derived astrocytes, consistent with the previous report that the majority of NeuN− cells are oligodendrocytes^21^. NeuN+ cells showed high similarity with adult brains, fetal brains, and iPSC-derived neurons. The fact that NeuN+ cells show high similarity with fetal brain may reflect extensive neurogenesis at midgestation, the developmental stage at which the fetal brain was obtained^22^.

We next interrogated the cell-type-specific nature of 3D chromatin structures such as compartments^23^ and FIREs^6,24^. We detected extensive compartment switching between NeuN+ and NeuN− cells: 4,333 regions (in 100kb resolution) switched from compartment A to B between NeuN− to NeuN+ cells, while 2098 regions switched from compartment B to A in NeuN+ to NeuN− cells. Importantly, genes located in compartments that switch from A to B between NeuN− and NeuN+ were highly expressed in oligodendrocytes and astrocytes, while those that switch from B to A in NeuN− to NeuN+ were highly expressed in neurons, suggesting that the difference in chromosome conformation between NeuN+ and NeuN− cells is associated with cell-type-specific gene regulation (Supplementary Fig. 3c).

**Fig. 1:**
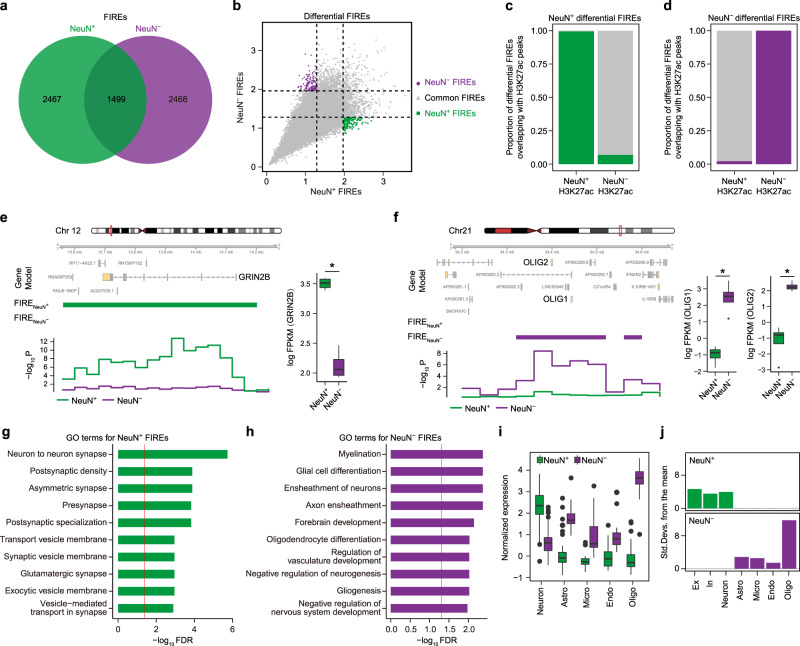
Differential FIREs are associated with cell-type-specific gene regulation. **a** The number of FIREs detected in NeuN+ and NeuN− cells. **b** Differential FIREs were identified in NeuN+ and NeuN− cells. **c**, **d** Differential FIREs overlap with differential H3K27ac peaks in the corresponding cell types. A neuronal gene, *GRIN2B*, is located in NeuN+ specific FIREs (**e**), while two oligodendrocytic genes, *OLIG1* and *OLIG2*, are located in NeuN− specific FIREs (**f**). FIREs and significance of FIRE scores in NeuN+ and NeuN− cells are depicted in green and purple, respectively. Boxplots in the right show expression levels of *GRIN2B* (FDR = 3.71e−12), *OLIG1* (FDR = 7.34e−26) and *OLIG2* (FDR = 8.06e−23) in NeuN+ (*n* = 4) and NeuN− (*n* = 4) cells. FPKM Fragments Per Kilobase of transcript per Million mapped reads. Center, median; box = 1st to 3rd quartiles (Q); minima, Q1 − 1.5 × interquartile range (IQR); maxima, Q3 + 1.5 × IQR. *FDR < 0.05 calculated by DESeq2 (two-sided Wald test). Source data are provided as a Source Data file. Gene ontology (GO) analysis for genes assigned to differential NeuN+ (**g**) and NeuN− (**h**) FIREs. The red line denotes FDR = 0.05. **i** Cellular expression levels of genes assigned to differential NeuN+ and NeuN− FIREs. Center, median; box = Q1−Q3; minima, Q1 − 1.5 × IQR; maxima, Q3 + 1.5 × IQR. Neurons, *n* = 131; astrocytes (Astro), *n* = 62; microglia (Micro), *n* = 16; endothelial (Endo), *n* = 20; oligodendrocytes (Oligo), *n* = 38. Source data are provided as a Source Data file. **j** Genes assigned to differential NeuN+ and NeuN− FIREs are enriched in neurons and glia, respectively. Ex excitatory neurons, In inhibitory neurons.

FIREs represent regions that act as interaction hubs, containing active enhancers that show high tissue specificity^6^. They are also enriched for genome-wide association study (GWAS) variants and exhibit variability across individuals^25,26^. We therefore compared FIREs in NeuN+ and NeuN− cells to identify how local chromatin architecture differs among these major brain cell types. We detected 3966 and 3967 FIREs in NeuN+ and NeuN− cells, respectively (Fig. 1a and Supplementary Data 1)^24^. Among them, 1248 FIREs were shared between NeuN+ and NeuN− cells based on the coordinate-level overlap and similarities in FIRE scores (hereafter referred to as common FIREs, “Methods”, Supplementary Data 1). Genes located in common FIREs were involved in housekeeping functions (e.g., microtubule polymerization, cell junction assembly) as well as pathways that involve both neurons and oligodendrocytes (e.g., main axon, Supplementary Data 1).

To further investigate how FIREs are associated with cell-type-specific gene expression, we used a stringent statistical cutoff to define differential FIREs on the basis of FIRE scores (“Methods”), detecting 287 differential FIREs between NeuN+ (145) and NeuN− (142) cells (hereby referred to as NeuN+ and NeuN− FIREs, respectively, Fig. 1b, Supplementary Data 2). Since previous reports have suggested that FIREs are closely linked to cell-type-specific enhancers^6^, we intersected differential FIREs with differential H3K27ac peaks between NeuN+ and NeuN− cells^27^. We found that the majority of NeuN+ and NeuN− FIREs overlapped with NeuN+ and NeuN− differential H3K27ac peaks, respectively, displaying remarkable cell-type-specificity (Fig. 1c, d). We next examined whether differential FIREs overlap with cell-type-specific marker genes. Indeed, NeuN+ FIREs overlapped with neuronal genes that were enriched for synaptic function (Fig. 1g), while NeuN− FIREs overlapped with genes involved in myelination, glial differentiation, and oligodendrocyte differentiation (Fig. 1h). Salient examples include *GRIN2B*, which overlaps with a NeuN+ FIRE, and *OLIG1* and *OLIG2*, which overlap with a NeuN− FIRE (Fig. 1e, f, Supplementary Fig. 4). We further checked cellular expression profiles of genes assigned to differential FIREs using single-cell (sc)RNA-seq data^10^. As expected, NeuN+ and NeuN− FIRE-associated genes were mainly enriched in neurons and glia, respectively (Fig. 1i, j). In particular, NeuN− FIRE-associated genes were most highly expressed in oligodendrocytes, confirming that NeuN− fraction is enriched for oligodendrocytes^21^.

Super-FIREs represent a small proportion of FIRE clusters with the most significant local interaction frequency^6^. Super-FIREs are thought to have a strong gene regulatory potential and often overlap with super enhancers^6^. We therefore identified super-FIREs in NeuN+ and NeuN− cells (“Methods”) and found that they also displayed substantial cellular specificities. Only 9 super-FIREs were detected in both NeuN+ and NeuN− cells, leaving 253 and 157 cell-type-specific super-FIREs in NeuN+ and NeuN− cells, respectively (Supplementary Fig. 5a, Supplementary Data 2). Over 95% of super-FIREs overlapped with differential H3K27ac peaks and all super-FIREs overlapped with promoters, indicating that they have a particularly strong cell-type-specific regulatory impact (Supplementary Fig. 5b, c). In line with these findings, super-FIREs were tightly coupled with cell-type-specific gene expression. NeuN+ super-FIREs overlapped with genes functioning in synapses and ion gated channels that were highly expressed in neurons, while NeuN− super-FIREs overlapped with genes involved in cell adhesion, myelination, and ensheathment of neurons, with high expression in glia (Supplementary Fig. 5d-f, 6). Taken together, these analyses show that differential FIREs and super-FIREs are strongly associated with cell-type-specific gene regulation in the nervous system.

### Characteristics of chromatin interactions in NeuN+ and NeuN− cells

**Fig. 2:**
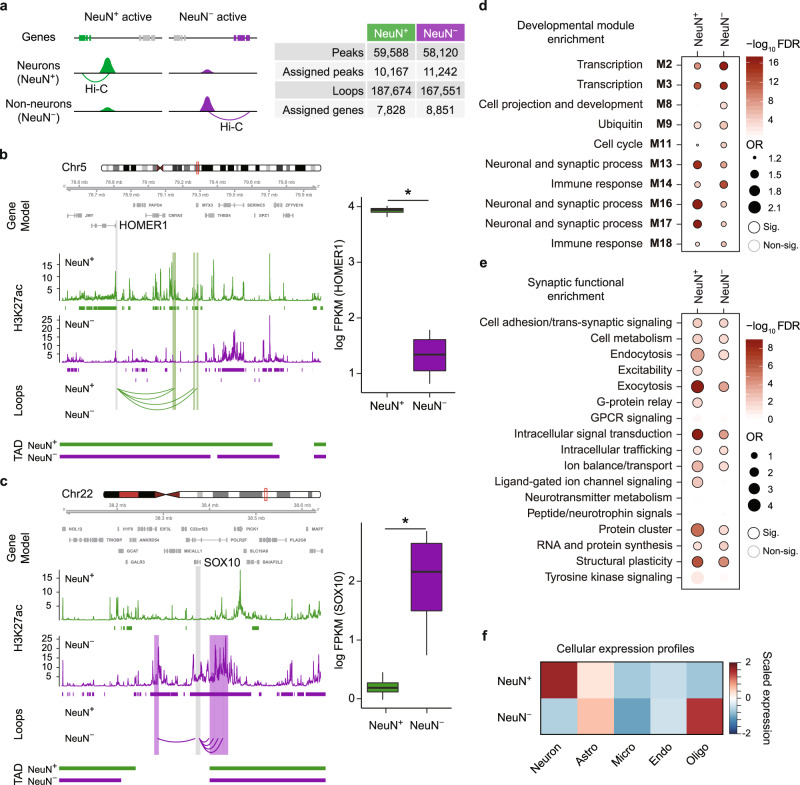
Enhancer–promoter interactions in NeuN+ and NeuN− cells. **a** (Left) cell-type-specific regulatory networks were built by linking genes to NeuN+ and NeuN− specific H3K27ac peaks via Hi–C interactions in NeuN+ and NeuN− cells, respectively. (Right) The number of cell-type-specific peaks and their assigned genes in NeuN+ and NeuN− cells is described. A neuronal gene, *HOMER1*, is engaged with NeuN+ specific H3K27ac peaks via loops in NeuN+ cells (**b**), while an oligodendrocyte gene, *SOX10*, is engaged with NeuN− specific H3K27ac peaks via loops in NeuN− cells (**c**). The regions that interact with the gene promoter (gray) are highlighted in green (NeuN+) and purple (NeuN−), respectively. Boxplots in the right show expression levels of *HOMER1* (FDR = 7.26e−32) and *SOX10* (FDR = 1.88e−49) in NeuN+ (*n* = 4) and NeuN− (*n* = 4) cells, respectively. FPKM Fragments Per Kilobase of transcript per Million mapped reads. Center, median; box = Q1–Q3; minima, Q1 − 1.5 × IQR; maxima, Q3 + 1.5 × IQR. *FDR < 0.05 calculated by DESeq2 (two-sided Wald test). Source data are provided as a Source Data file. **d** Genes assigned to NeuN+ specific peaks are enriched for synaptic co-expression modules, while genes assigned to NeuN− specific peaks are enriched for co-expression modules involved in transcriptional regulation and immune response during neurodevelopment. Significant enrichment (Sig.), FDR < 0.05. Fisher’s exact test was used for statistics analysis. OR, odds ratio. **e** Genes assigned to NeuN+ specific peaks are more highly enriched for synaptic functions such as exocytosis, intracellular signal transduction, protein cluster and structural plasticity than genes assigned to NeuN− specific peaks. Sig., FDR < 0.05. Fisher’s exact test was used for statistics analysis. **f** Genes assigned to NeuN+ specific peaks are highly expressed in neurons, while genes assigned to NeuN− specific peaks are highly expressed in oligodendrocytes and astrocytes. Astro astrocytes, Micro microglia, Endo Endothelial, Oligo oligodendrocytes.

We next identified promoter-anchored chromatin loops in NeuN+ and NeuN− cells to examine how chromatin interactions are associated with intricate regulation of cell-type-specific expression profiles. We detected 187,674 and 167,551 promoter-based interactions^3,4^ from NeuN+ and NeuN− cells, respectively (Fig. 2a). Over 75% of promoter-based interactions were detected within TADs (Supplementary Fig. 7a). Roughly 37% of interactions occurred between enhancers and promoters and ~23% of interactions occurred between two different promoters (Supplementary Fig. 7b). We infer that the promoter–promoter interactions are likely attributed to transcription factors (TFs) of coregulated genes. A substantial fraction of chromatin interactions were distal, as ~50% of chromatin interactions brought two genomic regions linearly separated by 320 kb or more into close proximity (Supplementary Fig. 7c) and captured complex enhancer–promoter interactions. For example, we found that the majority of promoters interact with more than one enhancer (Supplementary Fig. 7d), consistent with the previous findings that multiple enhancers can interact with one promoter^3,7^. Notably, the number of enhancers that interact with promoters was strikingly correlated with gene regulation, as gene expression linearly increased with the number of physically interacting enhancers up to a total of 10 or more (Supplementary Fig. 7e)^3,28^.

We next leveraged chromatin states predicted by ChromHMM^29^ to delineate the epigenetic properties of the genomic regions that interact with promoters (Supplementary Fig. 7f). As expected, promoters often interact with active chromatin features such as other transcription start sites (TSS, 1_TssA and 2_TssAFlnk) and enhancers (6_EnhG and 7_Enh). However, it is of note that a significant proportion of promoters also interact with bivalent marks (10_TssBiv, 11_BivFlnk and 12_EnhBiv), suggesting that the associated regions are poised to be activated upon stimulation. Together, our results confirmed that gene expression was coordinately controlled by physical interactions of promoters with enhancers, indicative of transcriptional regulators that underlie cell-type-specific gene regulation. So, we implemented GimmeMotifs^30^ to evaluate differential TF motif enrichment at cell-type-specific enhancers that interact with promoters in NeuN+ and NeuN− cells. TFs involved in neuronal fate commitment, including ZBTB18, SMARCC1, TBR1, and NEUROD2, were enriched in NeuN+ cells (Supplementary Fig. 7g). Conversely, TF motifs for SOX2, SOX3, SOX4, SOX6, and SOX9 were enriched in NeuN− cells (Supplementary Fig. 7g). NeuN− distal enhancers were also enriched for motifs for the IRF family, such as IRF4, IRF7, IRF8, and IRF9 (Supplementary Fig. 7g), which contains key regulators of neural immune pathways expressed in glial cells^31^.

To further identify how enhancer–promoter interactions regulate cellular expression profiles, we next overlapped chromatin interactions with cell-type-specific enhancers (“Methods”). We were able to assign 10,167 and 11,242 cell-type-specific H3K27ac peaks to 7828 and 8851 genes via chromatin interactions in NeuN+ and NeuN− cells, respectively (Fig. 2a, Supplementary Data 3). Linking cell-type-specific enhancers to cell-type-specific loops revealed specific aspects of chromatin architecture regulating cell-type-specific gene expression. For example, a gene that encodes a synaptic scaffolding protein, *HOMER1*^32^, was engaged in NeuN+ specific peaks and loops and its expression was significantly higher in NeuN+ cells compared with NeuN− cells (Fig. 2b). In contrast, a glial gene, *SOX10*^33^, was engaged in NeuN− specific H3K27ac peaks and Hi–C loops, and had higher expression in NeuN− cells than in NeuN+ cells (Fig. 2c). In line with these findings, NeuN+ enhancer–promoter interactions were enriched for synaptic and axonal genes, whereas NeuN− enhancer–promoter interactions were associated with actin-based motility (Supplementary Data 3). Moreover, genes assigned to NeuN+ specific peaks were enriched in the synaptic co-expression modules during neurodevelopment^34^ (Fig. 2d). Within a synapse, they were involved in specialized functions including exocytosis, intracellular signal transduction, and synaptic plasticity^35^ (Fig. 2e). Lastly, genes assigned to NeuN+ specific peaks were more highly expressed in neurons, while those assigned to NeuN− specific peaks were highly expressed in oligodendrocytes and astrocytes (Fig. 2f), demonstrating the tight relationship between cell-type-specific chromatin architecture and expression signature.

### Enhancer–promoter interactions in glutamatergic and GABAergic neurons

Single-cell expression profiles have demonstrated a remarkable transcriptional diversity within neuronal subtypes^10^. We therefore used H3K27ac peaks defined in purified Glu and MGE-derived GABA (hereafter referred to as GABA) neurons^18^ to deconvolute NeuN+ chromatin interactions into two major neuronal subtypes. We obtained 45,911 Glu-specific and 32,169 GABA-specific H3K27ac peaks and assigned them to 6234 and 4342 genes, respectively (Supplementary Fig. 8a, Supplementary Data 4). These genes showed a remarked level of neuronal specificity. Genes assigned to Glu peaks were highly expressed in excitatory neurons, such as layers (L)2/3 pyramidal neurons (Ex1) and L5/6 corticothalamic projection neurons (Ex7), while genes assigned to GABA peaks were highly expressed in inhibitory neurons, such as parvalbumin-expressing GABA interneurons (In6, Supplementary Fig. 8b, the definition of Ex1–8 and In1–8 neuronal subtypes is available in Supplementary Table 2). *GRIK4*, a gene that encodes an ionotropic class of glutamate receptor^36^, displayed complex chromatin interactions with multiple Glu peaks. In contrast, *GAD1*, a gene that encodes a well-known cellular marker for inhibitory neurons^37^, was engaged in GABA peaks via chromatin interactions (Supplementary Fig. 8c). Collectively, we established neuronal subtype-specific gene regulatory relationships by integrating Glu- and GABA-specific peaks with the NeuN+ (neuronal) chromatin interaction profiles defined here.

### Cell-type-specific nature of AD-associated epigenetic dysregulation

**Fig. 3:**
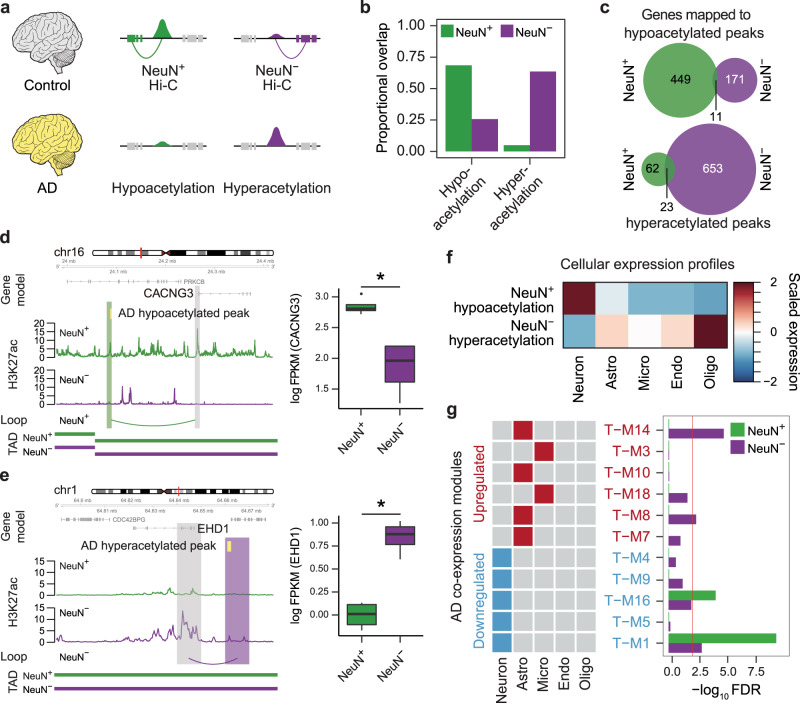
Cell-type-specific nature of epigenetic dysregulation in AD. **a** We built AD-associated gene regulatory networks by linking genes to hypoacetylated (hypo) and hyperacetylated (hyper) peaks in AD via Hi–C interactions in NeuN+ and NeuN− cells. **b** AD-associated hyperacetylated peaks were largely active in NeuN− cells, while AD-associated hypoacetylated peaks are largely active in NeuN+ cells in neurotypical controls. **c** The number of genes mapped to AD-associated hyperacetylated (top) and hypoacetylated (bottom) peaks via Hi–C interactions in NeuN+ and NeuN− cells. *CACNG3* is linked to an AD-associated hypoacetylated peak (marked in yellow) in NeuN+ cells (**d**), while *EHD1* is linked to an AD-associated hyperacetylated peak (marked in yellow) in NeuN− cells (**e**). *CACNG3*/*EHD1* promoter is highlighted in gray and its interacting regions are highlighted in green and purple for NeuN+ and NeuN− cells, respectively. Boxplots in the right show expression levels of *CACNG3*/*EHD1* in NeuN+ (*n* = 4) and NeuN− (*n* = 4) cells. FPKM Fragments Per Kilobase of transcript per Million mapped reads. Center, median; box = Q1–Q3; minima, Q1 − 1.5 × IQR; maxima, Q3 + 1.5 × IQR. *FDR < 0.05 (FDR = 2.81e−9 for *CACNG3* and FDR = 0.004 for *EHD1*), calculated by DESeq2 (two-sided Wald test). Source data are provided as a Source Data file. **f** NeuN+ hypoacetylated genes are highly expressed in neurons, while NeuN− hyperacetylated genes are highly expressed in glia. **g** NeuN− hyperacetylated genes are enriched in astrocyte-specific co-expression modules (T-M14 and T-M8) that are upregulated in AD. NeuN+ hypoacetylated genes are enriched in neuronal co-expression modules (T-M1, T-M16) that are downregulated in AD. Fisher’s exact test was used for statistical analysis. The red line denotes FDR = 0.01. Astro astrocytes, Micro microglia, Endo endothelial, Oligo oligodendrocytes.

We next used the cell-type-specific gene regulatory relationship to refine cell-type-specific aspects of disease vulnerability. Studies in AD have revealed changes in gene regulation manifested by differences in H3K27ac in bulk tissue^38^, but how this relates to cell-type-specific vulnerability is not known. To this end, we attempted to deconvolve the cell-type-specificity of epigenetic dysregulation detected in the AD brain tissue^38^ by overlapping AD-associated hyperacetylated and hypoacetylated H3K27ac peaks with NeuN+ and NeuN− peaks (Fig. 3a). We detected that AD-associated hyperacetylated peaks were largely active in NeuN− cells, while AD-associated hypoacetylated peaks were largely active in NeuN+ cells in neurotypical controls (Fig. 3b). To decipher the biological impact of AD-associated epigenetic dysregulation, we annotated these AD-associated hyperacetylated and hypoacetylated H3K27ac peaks using chromatin interaction profiles. We were able to link hypoacetylated peaks in AD to 460 genes using NeuN+ Hi–C data and hyperacetylated peaks in AD to 676 genes using NeuN− Hi–C data (hereby referred to as NeuN+ hypo- and NeuN− hyperacetylated genes, respectively, Fig. 3c, Supplementary Data 5). NeuN+ hypoacetylated genes include *CACNG3*, whose promoter formed a loop with a peak, which was preferentially active in NeuN+ cells and was hypoacetylated in postmortem AD brain. *CACNG3* encodes a voltage-gated calcium channel^39^ and had significantly higher expression in neurotypical NeuN+ cells compared to NeuN− cells (Fig. 3d). NeuN− hyperacetylated genes include *EHD1* (Fig. 3e), a gene involved in endocytic recycling with another AD-associated gene *BIN1*^40^. *EHD1* was both hyperacetylated and highly expressed in neurotypical NeuN− cells compared to NeuN+ cells. Importantly, the promoter of *EHD1* formed a loop with the AD hyperacetylated peak that was preferentially active in NeuN− cells. GO analysis demonstrated that NeuN+ hypoacetylated genes included synaptic genes while NeuN− hyperacetylated genes were involved in catalytic activity and glycoprotein binding (Supplementary Data 5). Cellular expression profiles also confirmed these findings, as NeuN+ hypoacetylated genes were highly expressed in neurons, while NeuN− hyperacetylated genes were highly expressed in oligodendrocytes, astrocytes, and endothelial cells (Fig. 3f). These results collectively suggest that in AD brains, many neuronal genes are downregulated due to their hypoacetylation in neurons, whereas many glial genes are upregulated due to their hyperacetylation in glia. To validate this prediction, we tested whether this epigenetic dysregulation in AD is associated with altered gene expression in AD by comparing NeuN− hyperacetylated and NeuN+ hypoacetylated genes with mRNA co-expression modules constructed from AD brains^41^ (Fig. 3g). Among 11 co-expression modules differently regulated in AD (see Supplementary Table 3 for module definition), NeuN− hyperacetylated genes were enriched in co-expression modules that were upregulated in AD (T-M8 and T-M14)^41^. These modules were involved in transcriptional regulation and cellular proliferation and were annotated as astrocyte-specific^41^. In contrast, NeuN+ hypoacetylated genes were enriched in co-expression modules that were downregulated in AD (T-M1 and T-M16)^41^. Both modules were associated with synaptic transmission. This result demonstrates that cell-type-specific epigenetic dysregulation in AD is coupled with gene expression changes.

### Genetic risk factors associated with AD converge onto microglial function

**Fig. 4:**
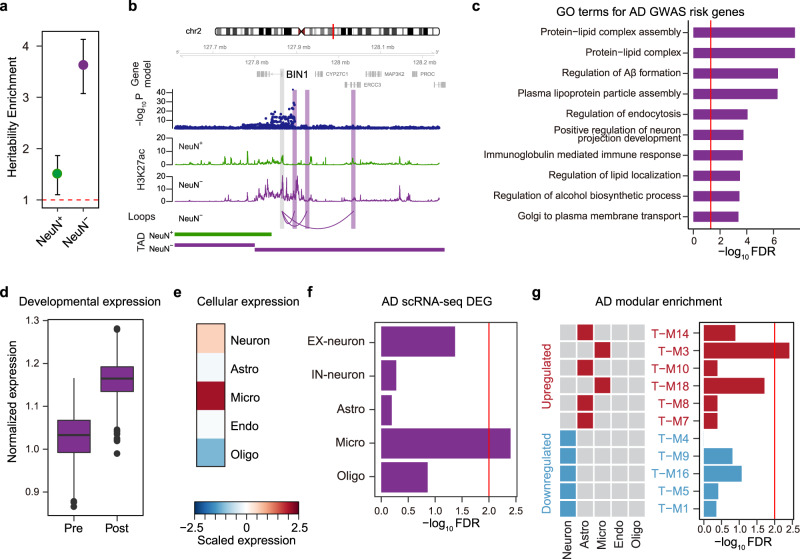
Identification and characterization of putative target genes of AD genetic risk factors by incorporating NeuN− chromatin interaction. **a** The SNP-based heritability enrichment of AD GWAS in differential NeuN+ and NeuN− peaks suggests glial enrichment. Enrichment ± standard error is depicted. The red broken line indicates heritability enrichment = 1. Source data are provided as a Source Data file. **b** *BIN1* promoter physically interacts with an AD GWS locus in a NeuN− specific manner. The regions that interact with *BIN1* promoter (marked in gray) are highlighted in purple. **c** GO analysis for GWAS-guided AD risk genes identified by NeuN− H-MAGMA. The red line denotes FDR = 0.05. **d** AD risk genes are highly expressed in postnatal brain samples compared with prenatal samples. Pre prenatal (*n* = 410); Post, postnatal (*n* = 453). *p* = 6.93e−193, calculated by Wilcoxon Rank Sum test. Center, median; box = Q1–Q3; minima, Q1 − 1.5 × IQR; maxima, Q3 + 1.5 × IQR. Source data are provided as a Source Data file. **e** AD risk genes are highly expressed in microglia. **f** AD risk genes are significantly enriched for genes differentially regulated in AD microglia. Fisher’s exact test was used for statistics analysis. The red line denotes FDR = 0.01. **g** AD risk genes are enriched in a microglial co-expression module that is upregulated in AD. Fisher’s exact test was used for statistical analysis. The red line denotes FDR = 0.01. Astro astrocytes, Micro microglia, Endo endothelial, Oligo oligodendrocytes.

Changes in gene expression or histone modifications may be a consequence or compensatory in disease and not necessarily causal. To link changes in chromatin and gene regulation to causal genetic factors, we next tested for enrichment of common genetic risk factors associated with AD by GWAS^42^. We reasoned that cell-type-specific annotation of the regulatory impact of genetic risk factors would provide support or refine our knowledge of causal mechanisms underlying AD, which to date heavily implicate neural immune/microglial mechanisms^43,44^. Therefore, we first performed linkage disequilibrium score regression (LDSC)^45^ analysis to determine the enrichment of AD-associated common genetic variants in NeuN− and NeuN+ H3K27ac peaks. AD SNP-based heritability was highly enriched in NeuN− enhancers (NeuN+ *p* = 0.205, NeuN− *p* = 8.41 × 10^−9^), suggesting that NeuN− gene regulatory relationships would be the most appropriate for refining biological insight from AD GWAS (Fig. 4a).

We next ran H-MAGMA^46^ built upon the NeuN− interactome to convert SNP-level association statistics into gene-level association statistics, thereby connecting non-coding variants to their cognate genes. This analysis identified 181 AD risk genes (FDR < 0.05) based upon their potential regulation by associated non-coding variants (“Methods”, Supplementary Data 6), many of which included well-known AD risk genes^47^. For instance, we found that AD genome-wide significant (GWS) SNPs interact with the promoter of *BIN1*, whose transcript level is increased in AD brains^48^ (Fig. 4b). Notably, the *BIN1* promoter was connected to multiple enhancers in NeuN−, but not in NeuN+ cells, demonstrating the highly cell-type-specific gene regulatory landscape within the locus. AD risk genes were enriched for amyloid−beta (Aβ) pathways, lipoprotein assembly, and immune processes (Fig. 4c, Supplementary Data 6). Consistent with previous studies^16,42,49^, these genes were highly expressed in the postnatal brain samples (Fig. 4d) and microglia (Fig. 4e). Notably, they were enriched for genes that are upregulated in microglia from postmortem AD brain^50^ (Fig. 4f). In line with this, we observed that AD risk genes that we identified here were enriched for a microglial co-expression module upregulated in AD postmortem brains (T-M3, Fig. 4g)^41^. This module is distinct from the module associated with epigenetic dysregulation in AD (Fig. 3g), in that the former represents microglia, while the latter is enriched in astrocytes. This is consistent with recent single-cell sequencing studies identifying genetic risk enrichment and gene expression changes within microglia in AD, accompanied by significant gene expression changes in other cells, including astrocytes^50–52^, which is where we observe epigenetic signature enrichment. Since genetic risk resides at the initiation of the causal chain in disease pathogenesis, this differential enrichment of genetic risk factors and epigenetic regulation underscores different expression signatures in AD with distinct cellular specificities and likely causal relationships.

### Refined cellular etiology of SCZ and BD

**Fig. 5:**
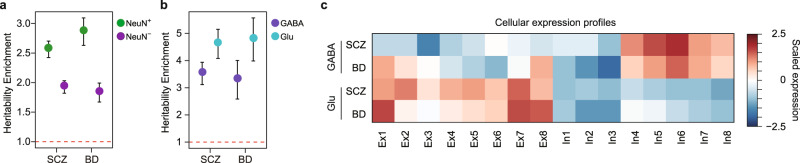
Comparison of SCZ and BD risk genes. **a** The SNP-based heritability enrichment of SCZ and BD GWAS in differential NeuN+ and NeuN− peaks demonstrates neuronal enrichment (SCZ NeuN+, FDR = 9.33e-24; SCZ NeuN−, FDR = 2.14e-16; BD NeuN+, FDR=3.94e-15; BD NeuN−, FDR=6.09e-07). Enrichment ± standard error is depicted. The red broken line indicates heritability enrichment = 1. Source data are provided as a Source Data file. **b** The SNP-based heritability enrichment of SCZ and BD GWAS in differential Glu and GABA peaks suggests that both Glu and GABA neurons are associated with the psychiatric disorders (SCZ GABA, FDR = 4.54e−9; SCZ Glu, FDR = 3.49e−10; BD GABA, FDR=1.10e−03; BD Glu, FDR = 3.63e−06). Enrichment ± standard error is depicted. The red broken line indicates heritability enrichment = 1. Source data are provided as a Source Data file. **c** Neuronal subtype expression profiles of SCZ and BD risk genes detected by GABA and Glu H-MAGMA.

In our previous study, we found that genes associated with psychiatric disorders display substantial molecular convergence within neurons, which was in stark contrast to neurodegenerative disorders like AD^46^. Indeed, LDSC (“Methods”) confirmed that SCZ and BD displayed strong SNP-based heritability enrichment in NeuN+ cells (Fig. 5a). We next hypothesized that enhancer-gene networks at a more refined cellular resolution (e.g. Glu and GABA neurons) would help elaborate molecular processes associated with these two major psychiatric disorders. Therefore, we assessed SNP-based heritability enrichment for SCZ and BD in Glu- and GABA-specific enhancers^18^. Notably, both disorders showed strong enrichment of SNP-based heritability in Glu- and GABA-specific enhancers, suggesting that both excitatory and inhibitory neurons may contribute to the genetic etiology of SCZ and BD (Fig. 5b). Since Hi–C data from neuronal subtypes is yet unavailable, we constructed Glu and GABA H-MAGMA maps by integrating Glu- and GABA-enhancers with NeuN+ Hi–C data, respectively (“Methods”), obtaining 753 SCZ risk genes from Glu (hereby referred to as Glu-SCZ genes) and 624 risk genes from GABA-specific regulatory interactions integrated via H-MAGMA (GABA-SCZ genes) (Supplementary Data 7). Glu-SCZ genes were implicated in synaptic organization, ion channel activity, and neuron projection, while GABA-SCZ genes were associated with dendrite, axon, and hormonal response (Supplementary Data 7). We also identified 143 and 101 BD candidate risk genes from Glu (Glu-BD genes) and GABA (GABA-BD genes) H-MAGMA, respectively (Supplementary Data 7). Glu-BD genes were enriched for neurogenesis, cell adhesion molecules, and synapses, while GABA-BD genes were enriched for transcriptional regulation and NMDA receptor activity (Supplementary Data 7). Neuronal subtype expression profiles of SCZ and BD risk genes identified by Glu and GABA H-MAGMA were evaluated using scRNA data obtained from excitatory (Ex1 to Ex8) and inhibitory (In1 to In8) neuronal subtypes^8^. Neuronal subtype enrichment showed a clear distinction between risk genes identified by Glu and GABA H-MAGMA, uncovering cellular and disease specificities that have not been described before (Fig. 5c). Glu-SCZ genes displayed widespread expression among many Glu neuronal subclasses, with relatively higher expression signatures in L3/4 neurons (Ex2) and L5/6 corticothalamic projection neurons (Ex7). Glu-BD genes showed a much stronger cell-type-specificity, with the highest expression signature in L2/3 cortical projection neurons (Ex1) and L5/6 corticothalamic projection neurons (Ex7-8). In interneurons, GABA-SCZ genes and GABA-BD genes showed similar enrichment for parvalbumin-expressing cells (In6). Given that genetic correlation between SCZ and BD is remarkably high (rg = 0.67)^46,53,54^, these findings suggest cellular substrates for molecular convergence (Ex7 and In6 are shared between two disorders) and divergence (Ex1 is BD-specific, while Ex2 is SCZ-specific) among two highly genetically correlated disorders.

## Discussion

Here we provide a high-resolution map of chromosome conformation from two major brain cell classes, neurons (NeuN+ cells) and glia (NeuN− cells, Supplementary Fig. 1). The genome-wide analysis of chromosome conformation in these two cell classes captures the major known elements of 3D architecture such as compartments, FIREs, and loops, addressing the roles and impact of these different hierarchical units in cell-type-specific gene regulation. We further refined maps of neuronal chromatin architecture by integrating Hi–C data from sorted neurons with H3K27ac peaks identified in two major neuronal subtypes, Glu and GABA neurons, which led to a successful delineation of neuronal subtype-specific regulatory relationships. We found that multiple architectural units that include compartments, FIREs, and chromatin loops display a remarked level of cellular specificity that is tightly coupled with gene regulation. We reasoned that these cell-type-specific gene regulatory networks would provide a window through which to understand the cellular etiology of brain diseases.

We first leveraged these networks to deconvolve epigenetic dysregulation in AD postmortem brain samples into the corresponding cell types. In their original work, Marzi et al.^38^ reported AD-associated H3K27ac peaks that are either hypo- or hyperacetylated in AD-affected individuals compared to age-matched low-pathology controls^38^ and linked these peaks to target genes on the basis of linear distance. We and others have shown that enhancers often regulate distal genes^4,15,16^, so this initial assignment is likely to be underpowered and inaccurate. Furthermore, these peaks were obtained from the brain homogenates, which would mask opposing interactions and obscure the cell-type-specific substrate of these changes. By developing neuronal and glial enhancer–promoter interaction maps, we not only accurately annotate these peaks with respect to their cognate genes, but also within their corresponding cell types. Notably, AD-associated hyperacetylation was enriched for glial enhancers that are associated with upregulation of astrocytic co-expression modules in AD, while AD-associated hypoacetylation was enriched for neuronal enhancers that potentially affect downregulation of a neuronal co-expression module in AD. These results highlight the importance of obtaining cell-type-specific epigenetic landscapes in diseases. This may reflect either the cellular composition changes (expansion of glia and neuronal death) or changes in regulatory landscape and cellular function (glial activation and decreased neuronal activity) in AD, which requires future investigation.

It is of note that while AD-associated hyperacetylation was linked to oligodendrocyte genes and astrocyte co-expression modules, genetic risk factors for AD were mapped onto a different glial cell type, microglia. These results are consistent with a newly emerging literature which indicates that different types of glia may contribute to the disease via different regulatory mechanisms^55–57^. In linking AD genetic risk factors to their cognate genes, we also recapitulated a previously found association between rs6733839 and *BIN1*. Nott et al. found that this SNP is causally implicated in the regulation of an AD risk gene, *BIN1*, in a microglia-specific manner^16^ as predicted by our analysis.

In contrast to AD in which multiple glial cells are implicated, previous work has indicated neurons as the central cell-type harboring genetic risk for the majority of psychiatric disorders^3,22,46,58–61^. However, given the strong genetic overlap between BD and SCZ^53,62^, we do not have much indication about specific biological pathways that are driving one disease versus the other. Here, we reasoned that neuronal subtype-specific gene regulatory networks would help refine the cellular etiology of disease and potentially identify discrete molecular neuropathology across psychiatric disorders. Indeed, when we applied Glu and GABA neuronal enhancer-gene networks to BD and SCZ, we were able to delineate discrete cellular substrates that may contribute to difference between BD (Ex1) and SCZ (Ex5), a distinction that has not been previously recognized. It is also of note that common genetic risk for SCZ and BD also indicated shared pathways coalescing onto parvalbumin-positive interneurons, paralleling what has been among the most robust pathologic findings in SCZ and BD^63,64^. However, we note that these findings need to be further verified by neuronal subtype-specific Hi–C data (e.g. Glu and GABA Hi–C). Moreover, CRISPR/Cas9-mediated genome engineering can be used to target risk genes’ enhancers to further investigate their roles in a neuronal subtype-specific manner. Our study provides a foundation for such future study by characterizing cell-type-specific 3D chromatin structure in the adult human brain, which we show can be used to improve our understanding of gene regulatory landscape in brain disorders.

## Methods

### Human tissue collection and nuclei isolation

Prefrontal cortex with no history of neurological disease was procured from tissue collections at the Department of Neuroscience at Yale University School of Medicine. Additional specimens were procured from the Brain and Tissue Bank at the University of Maryland. Tissue was collected after obtaining next of kin consent and with approval by the institutional review boards at the Yale University School of Medicine, the National Institutes of Health, and at each institution from which tissue specimens were obtained. Tissue specimens were handled in accordance with ethical guidelines and regulations for the research use of human brain tissue set forth by the NIH and the WMA Declaration of Helsinki. All available non-identifying information was recorded for each specimen (metadata available in Supplementary Table 1).

Procedures in preparation for flow cytometry (nuclei extraction, NeuN neuronal marker immunotagging using DAPI staining) for frozen never-fixed brain tissue specimens were previously described in detail^27,65^. Briefly, we dissected and homogenized 300 mg of frozen DLPFC tissue in lysis buffer (0.32 M Sucrose; 5 mM CaCl_2_; 3 mM Mg(Ace)_2_; 0.1 mM EDTA, pH8; 10 mM Tris-HCl, pH8; 1 mM DTT, 0.1% Triton X-100). Brain homogenate was fixed in 1% formaldehyde for 10 min prior to NeuN immunotagging^66^. Nuclei were isolated via ultracentrifugation in sucrose gradient. Isolated nuclei were subjected to NeuN immunotagging using 1:1000 anti-NeuN-488 (Millipore, MAB377X) and 1:1000 DAPI (Thermo Fisher Scientific, 62248) diluted in DPBS with 0.2% BSA. For each Hi–C assay, 2–5 × 10^6^ sorted NeuN+ or NeuN− nuclei were used. For each nuclear RNA-seq assay, ~5 × 10^4^ sorted nuclei were used.

### Hi–C library generation and data processing

Sorted cells were fixed in 1% formaldehyde for 10 min. Cross-linked DNA was then digested by HindIII (NEB, R0104). Digested chromatin ends were filled, marked with biotin-14-dCTP (Thermo Fisher, 19518-018), and ligated within the nucleus. DNA was sheared into 300–600-bp fragments (Covaris, M220), and biotin-tagged DNA was pulled down with streptavidin beads (Invitrogen, 65001) and ligated with Illumina paired-end adapters. The resulting Hi–C library was amplified by PCR (KAPA Biosystems HiFi HotStart PCR kit, KK2502), and sequenced by Illumina 50 bp paired-end sequencing.

The resulting Hi–C reads were mapped and filtered using hiclib (v.0.9)^67^. Filtered reads were binned at 10 kb, 40 kb, and 100 kb resolution to build a genome-wide contact matrix at a given bin size, which was subsequently normalized using Iterative Correction and Eigenvector decomposition. We then used 100 kb resolution matrices for compartment analysis, 40 kb for TAD analysis, and 10 kb for loop detection. Heatmaps of Hi–C contact matrices at 10 kb resolution were plotted using R package pheatmap (v.1.0.12) with scale=“column”.

### Comparison across multiple brain-derived Hi–C data

We used HiCRep (v.1.10.0)^19^ to measure reproducibility across biological replicates of Hi–C libraries using normalized Hi–C contact matrices at 40 kb resolution. HiCRep generates a smoothed contact matrix, and stratifies the matrix by the distance between the interacting regions of chromatin. From this stratified matrix, a SCC is defined, which provides a measure of similarity of genome-wide chromatin contacts across cell types.

HiCRep was also used to compare similarities between brain-derived genome-wide normalized chromatin contacts at 40 kb resolution. We compared Hi–C datasets derived from homogenized adult brain tissue (Adult brain)^3^, mature non-neuronal cells (NeuN−), mature neuronal cells (NeuN+), postmitotic neuron-enriched cortical plate from the fetal brain (Fetal brain CP)^4^, progenitor-enriched ventricular zone from the fetal brain (Fetal brain VZ)^4^, iPSC-derived neurons (iPSC Neuron)^15^ and iPSC-derived astrocytes (iPSC Astrocytes)^15^.

### Compartment calling

HiCExplorer (v.2.2.1.1)^68^ was used to call compartments from normalized genome-wide chromatin contact matrices at 100 kb resolution^68^. Principal component analysis (PCA) was performed using 4 eigenvectors. PC values were selected for each chromosome and correlated with gene density to determine compartments. The correlations between PC values from each Hi–C data were then used to compare similarity in compartments across cell types.

### TAD

We conducted TAD-level analysis as described previously^4^. In brief, we quantified the directionality index by calculating the degree of upstream or downstream (2 Mb) interaction bias of a given bin (40 kb), which was processed by a hidden Markov model (HMM) to remove hidden directionality bias.

### FIRE analysis

We used FIREcaller (v.1.10)^24^ to define FIREs and super-FIREs based on raw Hi–C contact matrices at 40 kb resolution. As FIREcaller defines FIREs based on a specific statistical threshold (*p* < 0.05 which corresponds to a FIRE score >1.56), NeuN+ or NeuN− specific FIREs defined by FIREcaller although displaying similar FIRE scores between two cell types, may be called significant only in one cell, as only one cell type exceeds the statistical cutoff (e.g. FIRE score = 1.6), while the other fails to reach the statistical cutoff (e.g. FIRE score = 1.5). Therefore, rather than using an all-or-none FIRE definition, we identified significantly differential FIREs based on the difference in FIRE scores between NeuN+ and NeuN−. NeuN+ differential FIREs were identified by obtaining the genomic regions that have NeuN+ FIRE scores greater than qnorm (0.975) and NeuN− FIRE scores lower than qnorm (0.9). NeuN− differential FIREs were defined in the opposite way; NeuN− FIRE scores<qnorm (0.9) & NeuN+ FIRE scores>qnorm (0.95). To link differential and super-FIREs to target genes, we intersected these differential FIREs and super-FIREs with gene promoters (defined as 2 kb upstream and 1kb downstream of TSS).

We also defined common FIREs, which are FIREs detected in both NeuN+ and NeuN− cells, and the difference of FIREs score between two cell types was less than 0.5. Common FIREs were then overlapped with promoters to identify common FIRE-associated genes. Since some of the common FIRE-associated genes also overlapped with NeuN+ and NeuN− FIREs, we removed those potential cell-type-specific genes to obtain common FIRE-associated genes.

### Loop calling

Promoter-based interactions were identified as previously described^3,4^. Briefly, we constructed background interaction profiles from randomly selected length- and GC content-matched regions to promoters. Using these background interaction profiles, we fit interaction frequencies into Weibull distribution at each distance for each chromosome using the *fitdistrplus*^69^ package in R. Significance of interaction from each promoter was calculated as the probability of observing higher interaction frequencies under the fitted Weibull distribution, and interactions with FDR < 0.01 were selected as significant promoter-based interactions. We overlapped promoter-based interactions with genomic coordinates of TADs, and found that the majority (~75%) of promoter-based interactions were located within the same TADs. We also applied Fit-Hi-C2 (v.2.0.7)^70^ with *-U 2000000 -L 20000 -r 10000 -p 2* to call loops.

### RNA-seq library generation and data processing

Nuclei (50,000 in DPBS) were sorted into 300 µl of Trizol (Fisher; Cat#: 15596018) such that the volume ratio of nuclei:Trizol was 1:3. Nuclei were lysed by pipetting 20 times in the Trizol solution on ice. RNA extraction was performed using the Zymo Directzol Microprep RNA Kit (Zymo; Cat#: R2062). RNA quality was evaluated using an Agilent Bioanalyzer 2100 with an RNA 6000 Pico Kit (Agilent; Cat#5067-1513). The RNA was then converted into cDNA and prepared into RNA-seq libraries using the SmarterStranded kit (Takara; Cat#634843). The libraries were size selected for an average fragment size of 300 bp using SPRI beads (Beckman Coulter Life Sciences; Cat#B23317). Library quality was assessed with a Qubit and Agilent Bioanalyzer 2100 using the Agilent High Sensitivity DNA Kit (Cat#5067-4626).

Once RNA-seq libraries were sequenced, we used FastQC (v.0.11.8)^71^ to check the quality of RNA-seq reads, and removed adapter with Cutadapt program (v.1.18)^72^. Next, clean reads were mapped to the human reference genome (Release 19 (GRCh37.p13)) from GENCODE database with HISAT2 (v.2.1.0)^73^ using default parameters. And, we assembled and quantified transcripts using StringTie (v.1.3.5)^74^. Differential analysis was done with DESeq2 (v.1.22.2)^75^. RNA-seq data from Glu and GABA neurons were obtained from a previously published study^18^.

### ChIP-seq data analysis

Differential H3K27ac peaks between NeuN+ and NeuN− cells were obtained from the previously published datasets^27^. We re-analyzed previously published H3K27ac ChIP-seq data from Glu and GABA neurons^18^ to define differential peaks between Glu and GABA neurons. We first used FastQC (v.0.11.8)^71^ to check the quality of ChIP-seq reads^18^. Next, we used Bowtie2 (v.2.3.4.3)^76^ with—very-sensitive to align reads to human reference genome build (Release 19 (GRCh37.p13)) from the GENCODE database. We removed duplicate reads using Picard (v.2.20.1, http://broadinstitute.github.io/picard/) MarkDuplicates function. We then called H3K27ac peaks using MACS2 (v.2.1.0.20150731)^77^ with—broad-cutoff 0.00001. Finally we used DiffBind (v.2.13.1)^78^ to analyze differentially binding regions between Glu and GABA ChIP-seq data.

### Motif analysis

To identify TFs that are involved in cell-type-specific distal regulation, we first extracted differentially accessible chromatin peaks by combining differential H3K27ac^27^ and ATAC-seq peaks^79^ that are brought to the promoters via chromatin loops. We then performed differential motif analyses on these cell-type-specific distal regulatory peaks using GimmeMotifs (gimme maelstrom) (v0.14.1)^30^ with the default settings.

### Gene Ontology Enrichment analysis

We used gProfileR2 (v.0.1.9)^80^ to identify GO terms that were overrepresented in particular gene sets such as genes assigned to common FIREs, differential FIREs, and super-FIREs. We set the arguments as following:

gost(input,organism = “hsapiens”,ordered_query = F,significant = T,evcodes = TRUE, user_threshold = 0.05, correction_method = “fdr”,sources = c(“GO”))$result

Top GO terms for super-FIRE-associated genes were highly redundant, which hindered the identification of biological pathways implicated for super-FIREs. We therefore applied REVIGO with default arguments (http://revigo.irb.hr/)^81^ to remove redundant GO terms.

To analyze functional enrichment of H-MAGMA genes, we used a previously reported rank-based approach^46^. This approach is a threshold-free method, because it does not require a specific FDR cutoff. Instead, biological pathways enriched in the top ranked genes are queried. Briefly, gProfileR (v.0.7.0)^80^ was used to perform GO analyses for ranked gene lists from H-MAGMA (v.1.08)^82^ for AD, SCZ, and BD GWAS:

gprofiler(input,organism = “hsapiens”, ordered_query = T, significant = T, max_*p*_value = 0.05, min_set_size = 15, max_set_size = 600, min_isect_size = 5, custom_bg = background gene set, correction_method = “fdr”, hier_filtering = “moderate”, include_graph = T, src_filter =“GO”)

custom_bg: H-MAGMA output genes overlapping with MCH regions were filtered (background gene set);

input: background gene set was ordered based on *p* value (Ranked gene list)

We used gProfileR to perform GO analysis for NeuN+ hypoacetylated and NeuN− hyperacetylated genes:

gprofiler(input,organism=“hsapiens”, ordered_query=F, significant=T, max_p_value=0.05, min_set_size=15, min_isect_size=5, correction_method=“fdr”, hier_filtering=“moderate”, include_graph=T, src_filter=“GO”).

### Cellular expression profile analysis

To quantify the significance of cellular expression of the genes assigned to cell-type-specific chromatin architecture (e.g., differential FIREs and loops), we used EWCE (v.1.3.0)^83^. In addition, cellular expression profiles of the disease risk genes were interrogated by plotting centered expression values for each cell type using the scRNA-seq data as described before^10,46^. Each neuronal subtype definition is provided in Supplementary Table 2.

### Module enrichment analysis

Developmental and synaptic modules were obtained from Parikshak et al.^34^ and Lips et al.^35^, respectively. We employed Fisher’s exact test to compare developmental and synaptic modules with genes engaged in NeuN+ and NeuN− enhancer–promoter interactions. AD-associated co-expression networks were obtained from Seyfried et al.^41^ (Supplementary Table 3).

AD co-expression modules were compared with (1) genes that were linked to hyper and hypoacetylated genes in AD and (2) AD-associated genes identified by H-MAGMA^46^ using Fisher’s exact test.

### Linking AD-associated epigenetic dysregulation to cognate genes

We downloaded AD-associated hyper- and hypoacetylated H3K27ac peaks from Marzi et al.^38^. Because these peaks were obtained from the brain homogenate that lacks cellular resolution, we overlapped them with NeuN+ and NeuN− differential peaks. Since we found that hyperacetylated peaks in AD significantly overlapped with NeuN− differential peaks, we used NeuN− loops to assign them to the target genes. On the other hand, hypoacetylated peaks in AD overlapped with NeuN+ differential peaks, so they were annotated to the target genes by NeuN+ loops.

### GWAS data

We downloaded the following GWAS summary datasets: BD (*n* = 20,352 cases; 31,538 controls)^84^, SCZ (*n* = 11,260 cases; 24,542 controls)^1^, and AD (*n* = 71,880 cases; 383,378 controls)^49^.

### LD score regression analysis

We implemented the LDSC software^45^ (v.1.0.0) to estimate the enrichment of SNP-based heritability for brain disorder GWAS in differential H3K27ac peaks between (1) NeuN+ and NeuN− cells and (2) Glu and GABA neurons. Genetic variants were annotated to differential H3K27ac peaks, and SNP-based heritability statistics were calculated using the GWAS summary statistics mentioned above. Enrichment statistics was calculated as the proportion of SNP-based heritability divided by the proportion of SNPs annotated to differential H3K27ac peaks.

### H-MAGMA input file generation

We generated H-MAGMA (v.1.08) input files that provide SNP-gene relationships based on chromatin interaction profiles from NeuN+ and NeuN− cells. Exonic and promoter SNPs were directly assigned to their target genes based on their genomic location. Intronic and intergenic SNPs were assigned to their cognate genes based on chromatin interactions with promoters and exons as previously described^46^. To provide SNP-gene relationships at a neuronal subtype resolution, we also generated Glu and GABA H-MAGMA input files. For example, we obtained SNPs that map onto Glu H3K27ac peaks, then SNPs active in Glu neurons were assigned to their genes by either genomic coordinates (when located in promoters and exons) or distal interactions (non-coding SNPs were mapped to their cognate genes via NeuN+ chromatin interactions). We used the same framework to obtain GABA SNP-gene relationships. Input files can be found in the github repository: https://github.com/thewonlab/NeuN.

### H-MAGMA

Based on the SNP-based heritability enrichment result, we used NeuN− H-MAGMA to infer AD risk genes and Glu/GABA H-MAGMA to infer SCZ and BD risk genes.

We set the arguments as following: magma_v1.08/magma—bfile g1000_eur –pval <GWAS summary statistics> use = rsid,p ncol = N—gene-annot <MAGMA input annotation file>—out <output file>.

g1000_eur denotes the reference data file for European ancestry population.

### Python and R environments

We used Python v.2.7 and R v.3.6.0.

### Reporting summary

Further information on research design is available in the Nature Research Reporting Summary linked to this article.

## Supplementary information

Supplementary Information

Description of Additional Supplementary Files

Supplementary Data 1

Supplementary Data 2

Supplementary Data 3

Supplementary Data 4

Supplementary Data 5

Supplementary Data 6

Supplementary Data 7

Reporting summary

## Data Availability

Hi–C data described in this manuscript is available through the PsychENCODE Knowledge Portal with the accession code of syn21760712. H3K27ac, ATAC-seq, and RNA-seq data from NeuN+ and NeuN− cells are available through syn4566010, GSE83345, and syn20545534, respectively. H3K27ac ChIP-seq data from Glu and GABA neurons are available through syn12034263. Human reference genome and gene definition was obtained from GENCODE and UCSC genome browser [http://genome.ucsc.edu]. All other relevant data supporting the key findings of this study are available within the article and its Supplementary Information files or from the corresponding authors upon reasonable request. Source data are provided with this paper. A reporting summary for this Article is available as a Supplementary Information file. Source data are provided with this paper.

## Source data

Source Data

## Code availability

All custom code used in this work is available in the following github repository: https://github.com/thewonlab/NeuN^85^.
