# Supplementary material for: Neuronal and glial 3D chromatin architecture informs the cellular etiology of brain disorders
Source: Nat Commun. 2021 Jun 25;12:3968. doi: 10.1038/s41467-021-24243-0 (PMC8233376; doi:10.1038/s41467-021-24243-0)
Supplement: Supplementary file 1 — Supplementary Information [file 41467_2021_24243_MOESM1_ESM.pdf]

Supplementary Information: Neuronal and glial 3D chromatin architecture informs the cellular etiology of brain disorders

I. Supplementary Tables

Supplementary Table 1. Metadata for samples and Hi-C libraries.

Hi-C library	Sample information	cis (filtered reads)	total (filtered reads)	cis ratio
HSB189 NeuN+	Neurotypical Male 36yr (Ancestry unknown)	408,825,167	518,035,101	0.79
HBS189 NeuN−		221,335,304	389,703,477	0.57
HBS277 NeuN+	Neurotypical Male 53yr (Caucasian)	252,224,585	328,763,010	0.76
HBS277 NeuN−		201,700,109	355,041,078	0.57
HBS106 NeuN+	Neurotypical Male 64yr (Ancestry unknown)	116,123,241	148,923,644	0.78
HBS106 NeuN−		174,635,894	270,901,173	0.64
HBS181 NeuN+	Neurotypical Male 44yr (Caucasian)	86,732,901	114,104,466	0.76
HBS181 NeuN−		57,617,646	82,727,024	0.65
NeuN+ pooled		863,905,894	1,109,826,221	0.78
NeuN− pooled		743,402,261	1,235,074,966	0.60

Supplementary Table 2. Neuronal subtype definition.

Neuronal subtype	Class	Molecular markers and cortical layer identity
Ex1	excitatory neuron	cortical projection neuron (layer 2/3)
Ex2		granule neuron (layer 3/4)
Ex3		granule neuron (layer 4)
Ex4		subcortical projection neuron (layer 4)
Ex5		subcortical projection neuron (layer 4)

Ex6		subcortical projection neuron (layer 5)
Ex7		corticothalamic projection neuron (layer 5/6)
Ex8		corticothalamic projection neuron (layer 6)
In1	inhibitory neuron	VIP+ RELN+ NDNF+
In2		VIP+ RELN- NDNF-
In3		VIP+ RELN+ NDNF-
In4		VIP- RELN+ NDNF+
In5		CCK+ nNOS+ CB+
In6		PV+ CRHBP
In7		SOM+ CB+ NPY+
In8		SOM+ nNOS+

SOM, somatostatin; NPY, neuropeptide Y; CB, calbindin; VIP,vasoactive intestinal peptide; RELN, reelin; nNOS, neuronal nitric oxide synthase; PV, parvalbumin or PVALB; CCK, cholecystokinin; NDNF, neuron-derived neurotrophic factor; CRHBP, corticotropin releasing hormone binding protein.

Supplementary Table 3. Eleven co-expression modules associated with AD (reported from Seyfried et al[1]).

**Modules**	**AD-association**	**Cell-type**	**Functions**
T-M1	Downregulated in AD	Neuron	synapse
T-M3	Upregulated in AD	Micro	immune system process
T-M4	Downregulated in AD	Neuron	metabolic process
T-M5	Downregulated in AD	Neuron	metabolic process
T-M7	Upregulated in AD	Astro	mRNA processing
T-M8	Upregulated in AD	Astro	transcription regulation
T-M9	Downregulated in AD	Neuron	mitochondrial electron transport

T-M10	Upregulated in AD	Astro	regulation of cell shape
T-M14	Upregulated in AD	Astro	transcriptional regulation
T-M16	Downregulated in AD	Neuron	synapse
T-M18	Upregulated in AD	Micro	cellular component disassembly

II. Supplementary Figures

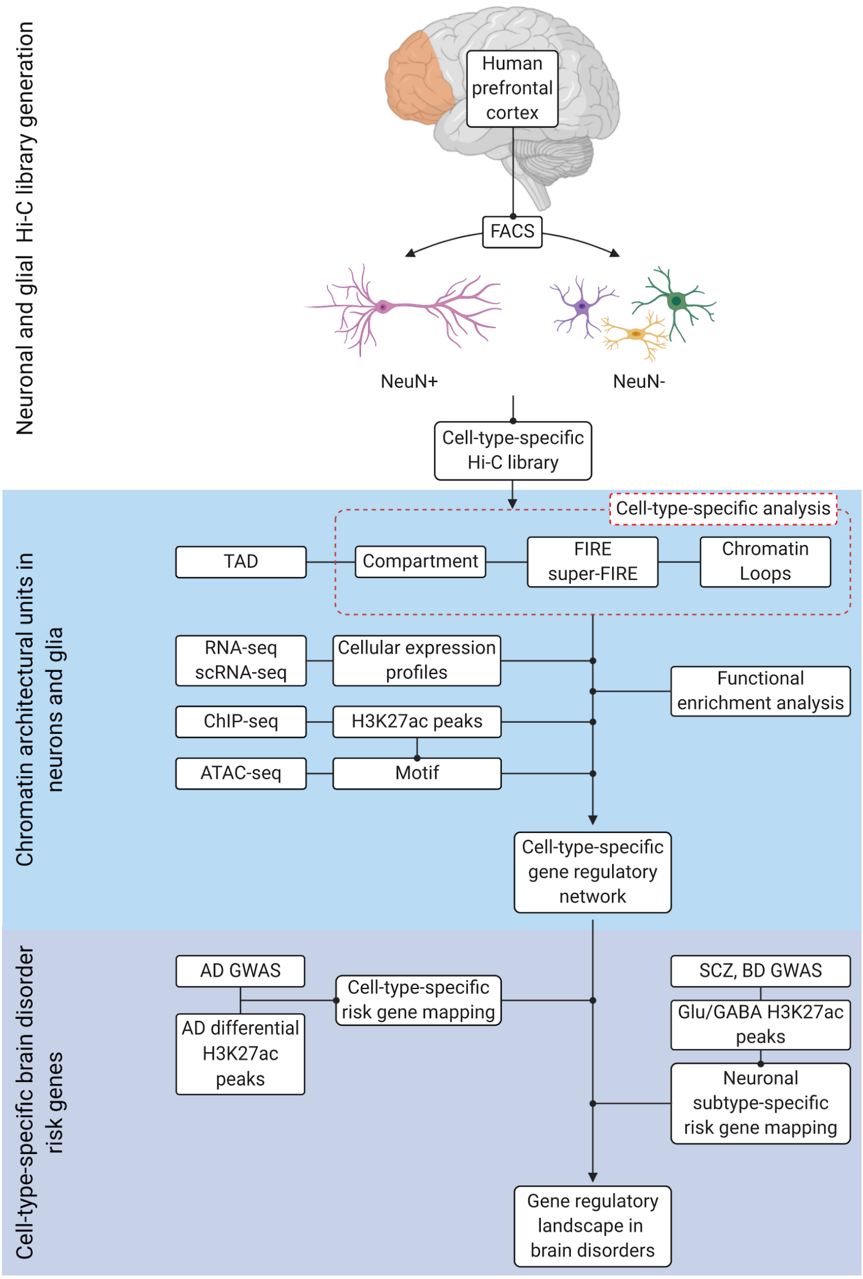

Supplementary Figure 1. Hi-C and integrative analysis pipeline. In step1, Hi-C libraries were generated from NeuN+ and NeuN− cells sorted from the human DLPFC. In step2, cell-type-specific architectural units such as compartment, FIREs, and chromatin loops were integrated with epigenomic and transcriptomic datasets to identify functional enrichments and define cell-type-specific gene regulatory networks. In step3, chromatin loops were used to characterize the impact of brain disorder genetic risk in a more cell-type-specific manner. This Figure was created with BioRender.com.

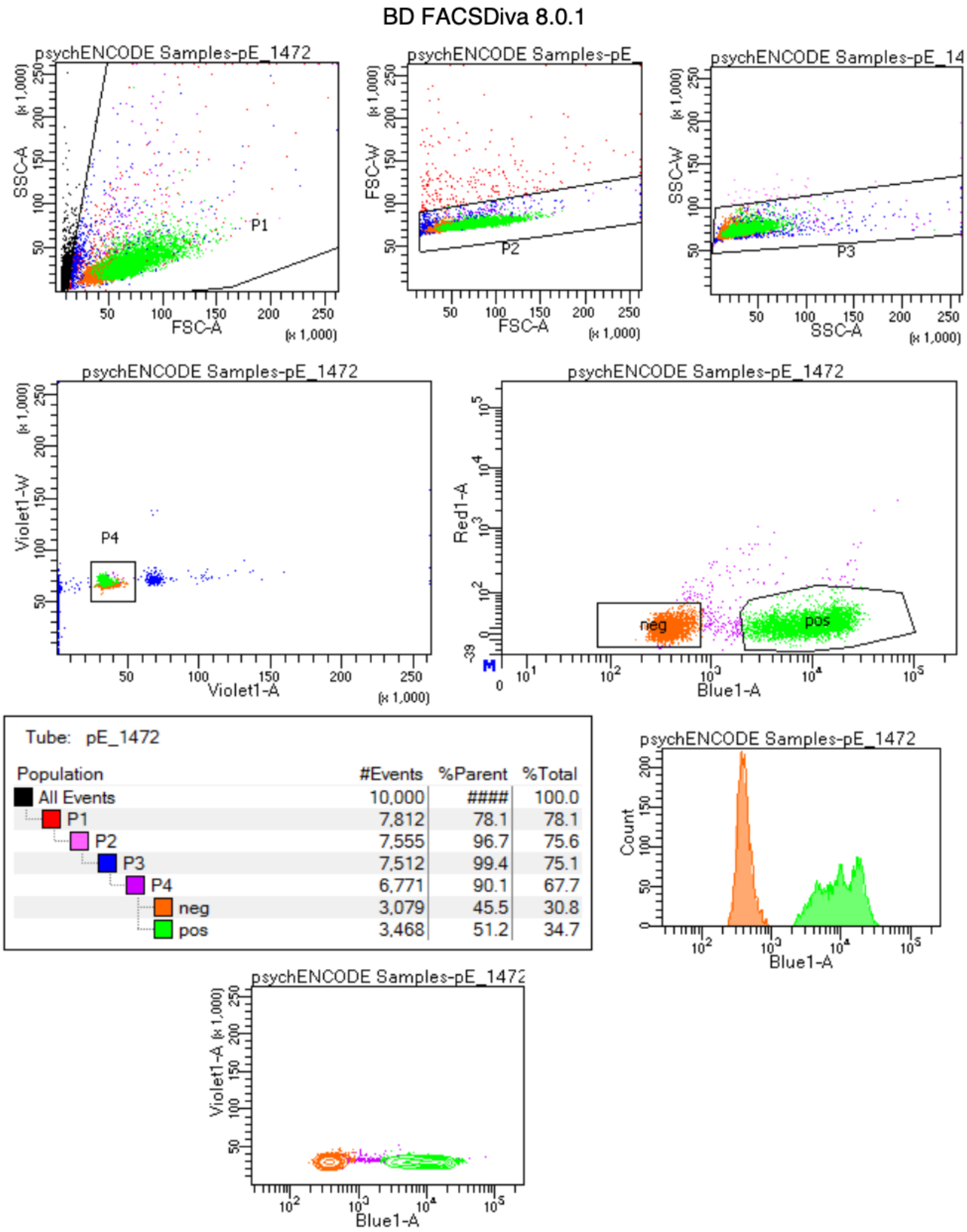

Supplementary Figure 2. Gating schematics used for fluorescence-activated nuclei sorting (FANS). Representative image depicting the sorting of NeuN+ and NeuN− nuclei from homogenized postmortem human brain PFC tissue. Forward scatter (FSC) and side scatter (SSC) gating is used to identify events of interest while excluding debris and clumps of nuclei. Nuclei are then gated by DAPI-staining to select for nuclei from cells not undergoing cell division. Finally, gating parameters are established to discriminately collect NeuN+ and NeuN− on the basis of Alexa-488 fluorescence.

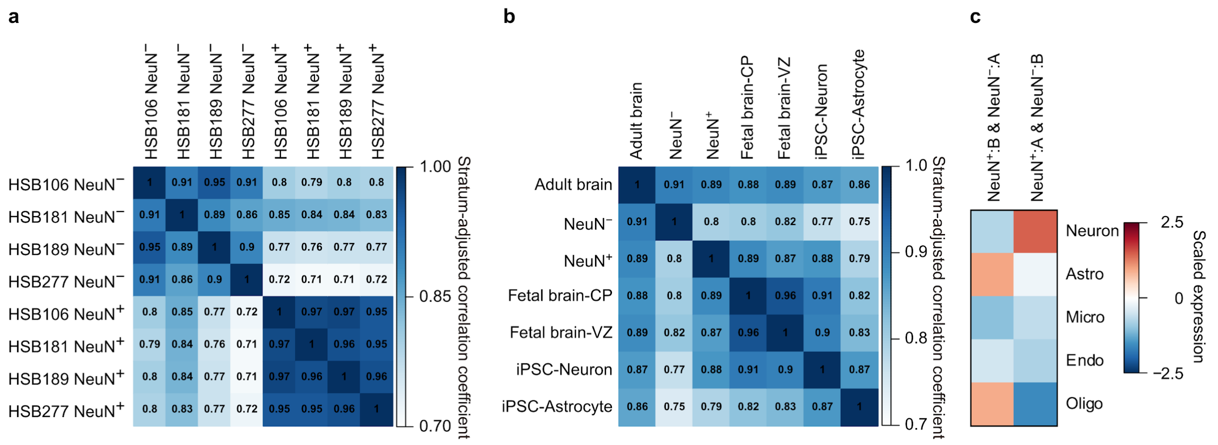

Supplementary Figure 3. Similarities across Hi-C datasets derived from brain-relevant tissues across developmental epochs and cell types. a-b. Stratum adjusted correlation coefficients analyzed by HiCRep[2] across NeuN+ and NeuN− Hi-C libraries (**a**) and brain-derived Hi-C data (**b**). It is of note that the result in (**b**) can be potentially confounded by batch effects. Moreover, HiCRep measures overall structural similarities at low resolution (40kb), which may not represent a refined view of cell-type-specificity. **c.** Cellular expression profiles of genes located in compartments that switch between NeuN+ and NeuN− cells. NeuN+:B & NeuN−:A, genes located in regions that undergo compartment switching from compartment B in NeuN+ cells to compartment A in NeuN− cells; NeuN+:A & NeuN−:B, genes located in regions that undergo compartment switching from compartment A in NeuN+ cells to compartment B in NeuN− cells. Astro, Astrocytes; Micro, Microglia; Endo, Endothelial; Oligo, Oligodendrocytes.

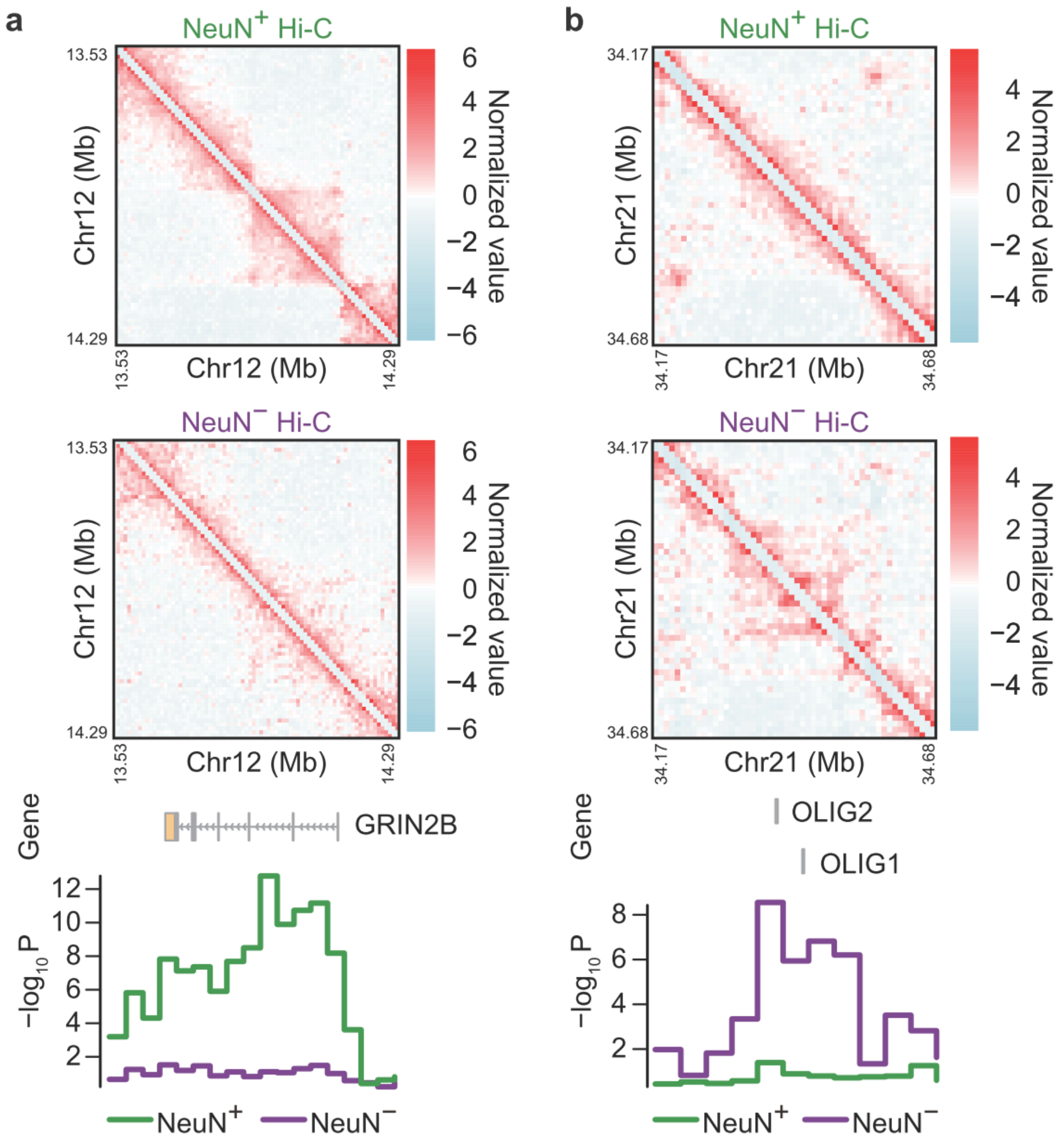

Supplementary Figure 4. Visualization of contact heatmaps corresponding to differential FIREs. a-b. Contact matrices and FIRE P-values for *GRIN2B* (**a**) and *OLIG1/2* (**b**) loci. Heatmaps represent 10kb normalized Hi-C contact matrices in NeuN+ (top) and NeuN− (middle) cells. FIRE P-values for NeuN+ and NeuN− cells are described in the bottom. Normalized value represents normalized contact frequency. The FIRE p-value was calculated by one-sided Z-test.

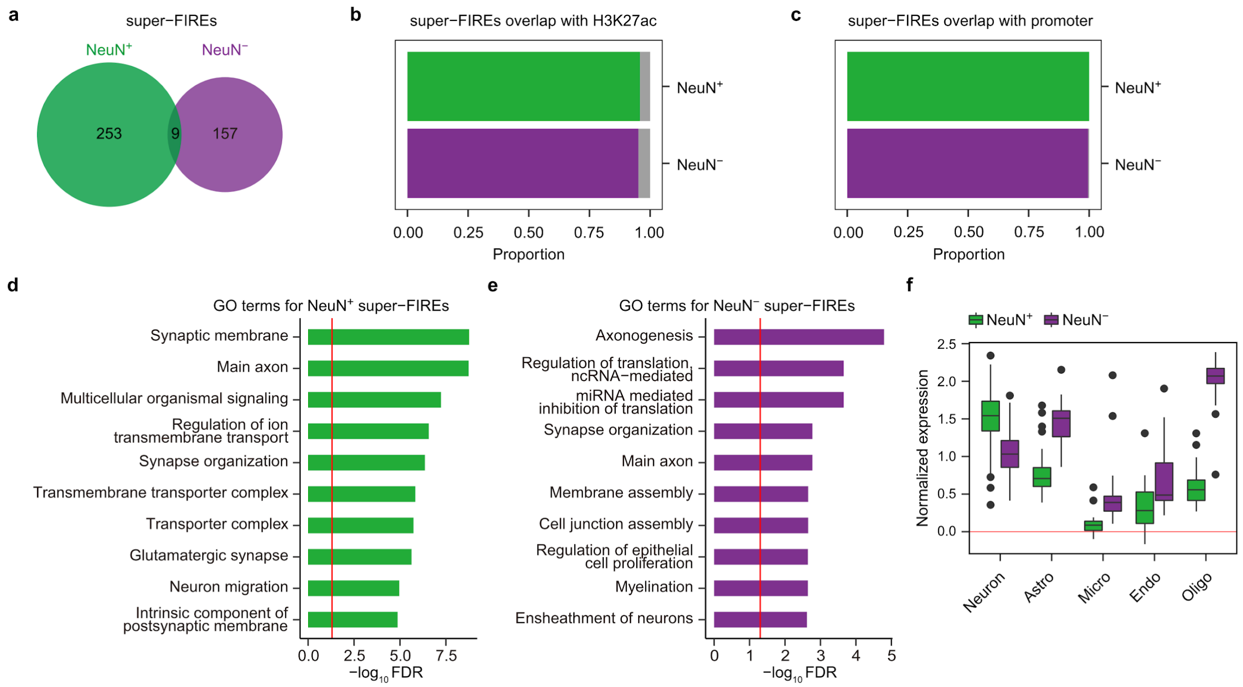

Supplementary Figure 5. Super-FIREs are associated with cell-type-specific gene regulation. a. Comparison between super-FIREs between NeuN+ and NeuN− cells. **b.** The majority of super-FIREs overlap with H3K27ac peaks, demonstrating their gene regulatory potentials. **c**. The majority of super-FIREs overlap with promoters. **d-e**. GO analysis for genes assigned to NeuN+ (**d**) and NeuN− (**e**) super-FIREs. The red line denotes FDR=0.05. **f**. Cell-type expression levels of genes assigned to NeuN+ and NeuN− super-FIREs indicate that NeuN+ super-FIREs overlap with genes highly expressed in neurons, while NeuN− super-FIREs overlap with genes highly expressed in glia. Neurons, n=131; Astrocytes (Astro), n=62; Microglia (Micro), n=16; Endothelial (Endo), n=20; Oligodendrocytes (Oligo), n=38. Center, median; box = Q1-Q3; minima, Q1 - 1.5 x IQR; maxima, Q3 + 1.5 x IQR. Astro, Astrocytes; Micro, Microglia; Endo, Endothelial; Oligo, Oligodendrocytes. Source data are provided as a Source Data file.

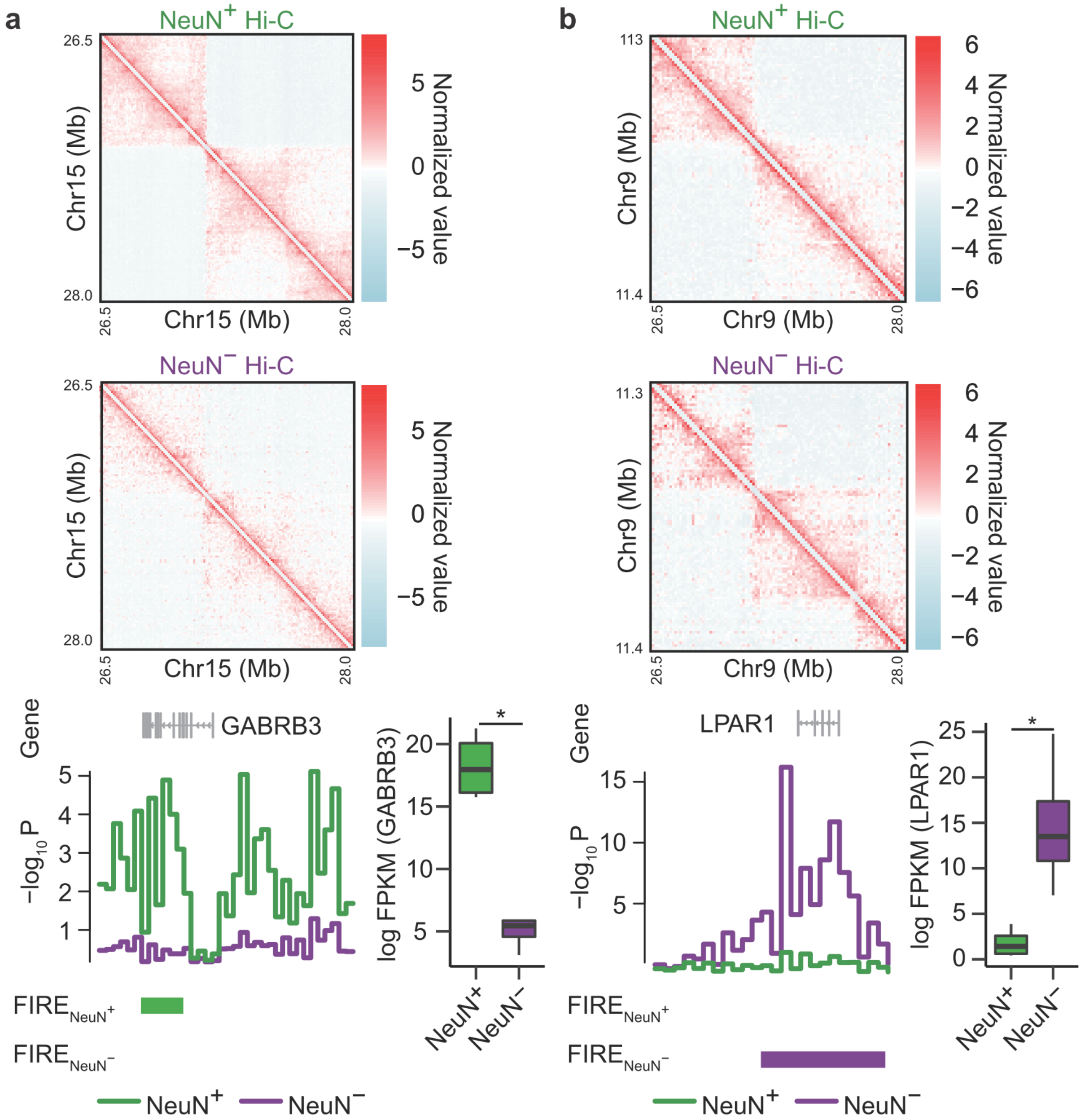

Supplementary Figure 6. Visualization of contact heatmaps corresponding to super-FIREs. a-b. An interneuronal gene, *GABRB3*, overlaps with a NeuN+ super-FIRE (**a**), while a glial gene, *LPAR1*, is located in a NeuN− super-FIRE (**b**). Heatmaps represent 10kb normalized Hi-C contact matrices in NeuN+ (top) and NeuN− (middle) cells. FIRE p-values and super-FIRE coordinates for NeuN+ and NeuN− cells are described in the bottom. The FIRE p-value was calculated by one-sided Z-test. Boxplots in the right show expression levels of *GABRB3* (FDR=1.12e-09) and *LPAR1* (FDR=7.19e-09) in NeuN+ (n=4) and NeuN− (n=4) cells. Normalized value represents normalized contact frequency. FPKM: Fragments Per Kilobase of transcript per Million mapped reads. Center, median; box = Q1-Q3; minima, Q1 - 1.5 x IQR; maxima, Q3 + 1.5 x IQR. *FDR<0.05 calculated by DESeq2 (two-sided Wald test). Source data are provided as a Source Data file.

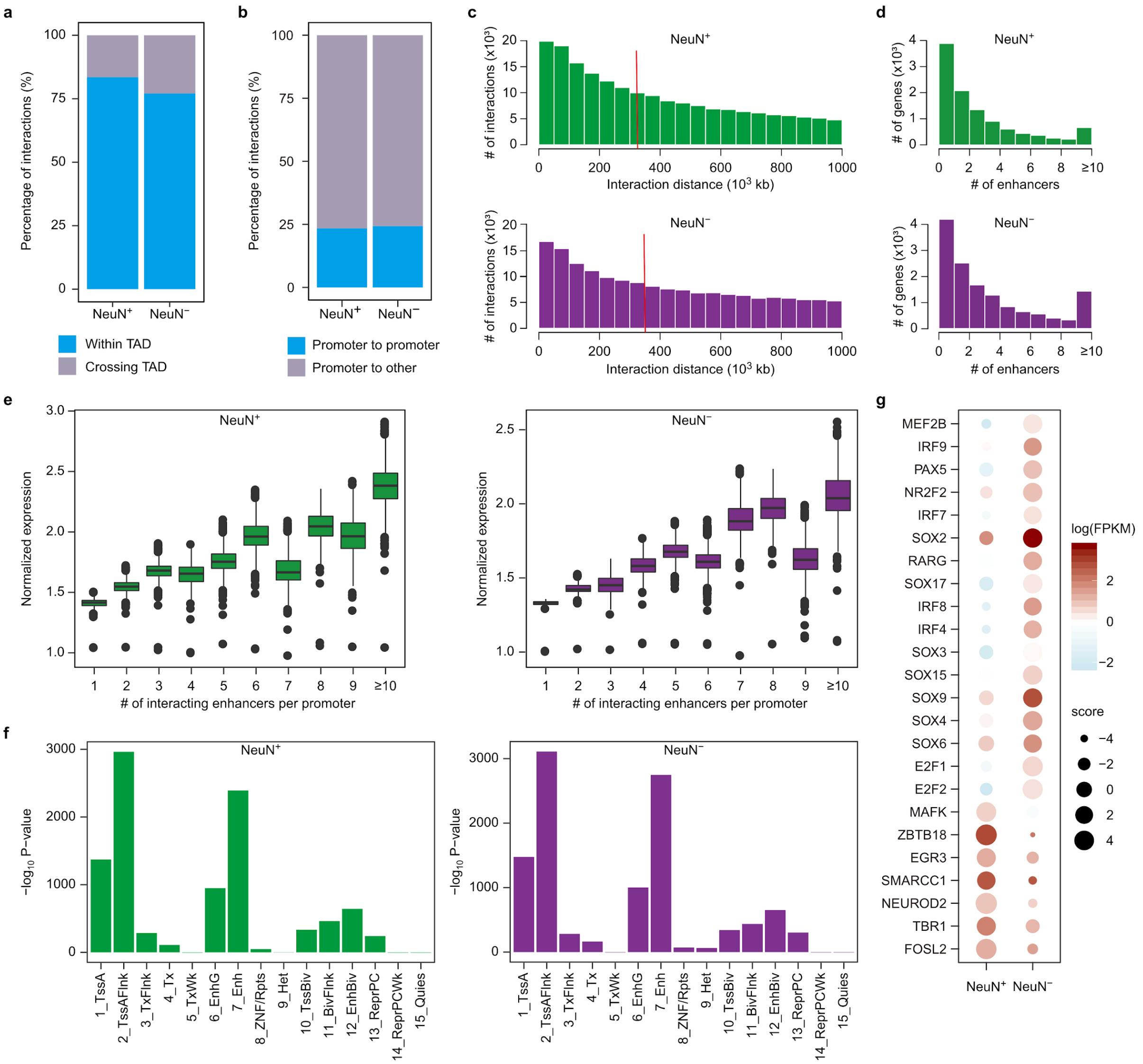

Supplementary Figure 7. **Characterization of physical chromatin interactions in NeuN+ and NeuN− cells**. **a.** Proportions of interactions occurring within TADs for NeuN+ and NeuN− cells. **b.** Proportions of promoter-promoter interactions in NeuN+ and NeuN− cells. **c.** Distribution of distance between interacting regions in NeuN+ (top) and NeuN− (bottom) cells. Red vertical lines represent average distance. **d.** A substantial fraction of promoters interact with more than one enhancer. **e.** The number of enhancers that interact with a given promoter linearly correlates with the target gene expression level (n=1,931). Center, median; box = Q1-Q3; minima, Q1 - 1.5 x IQR; maxima, Q3 + 1.5 x IQR. Linear regression (one-sided F-test) reveals a strong relationship between gene expression values and the number of interacting enhancers (NeuN+: $p=0.00049$, $r^2=0.77$; NeuN−: $p=0.0010$, $r^2=0.73$). Source data are provided as a Source Data file. **f.** Regions that interact with promoters are highly enriched in promoter and enhancer states, consistent with those representing functional promoter-promoter and enhancer-promoter loops. Epigenomic chromatin states were inferred using ChromHMM in the adult brain. **g.** Enrichment of consensus transcription factor (TF) motif sequences at NeuN+ and NeuN− open chromatin peaks that are engaged in enhancer-promoter interactions in NeuN+ and NeuN− cells, respectively. The size of each dot represents the degree of enrichment for each TF motif in each cell type, and the color of each dot represents TF expression levels.

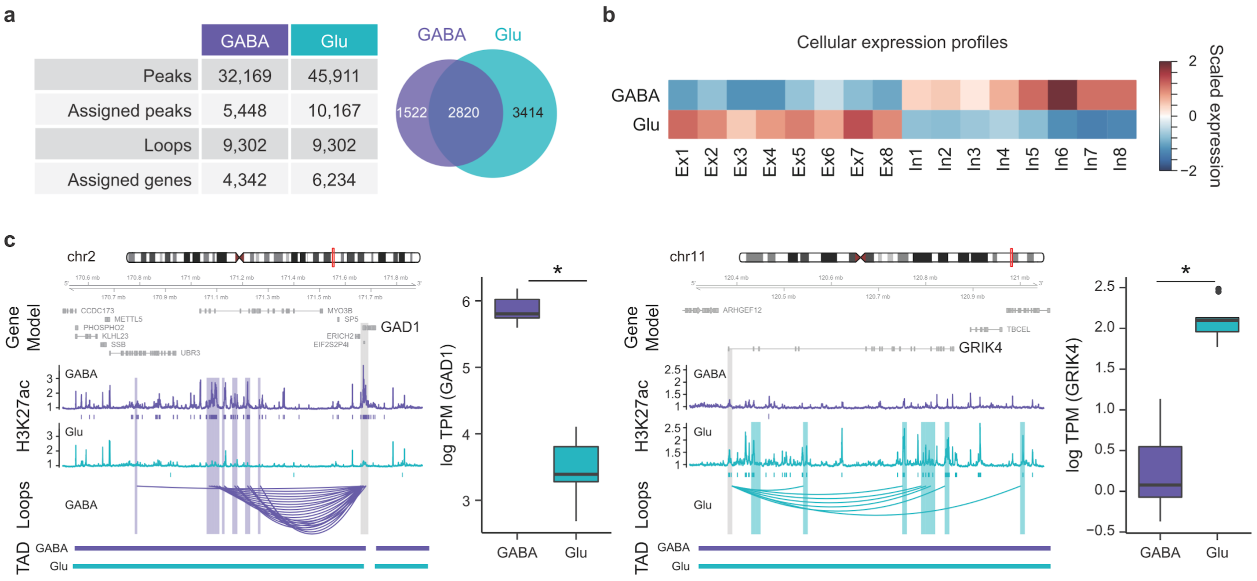

Supplementary Figure 8. **Comparison of enhancer–promoter interactions in Glu and GABA neurons**. **a**. (Left) The number of cell-type-specific peaks and their assigned genes in GABA and Glu neurons. (Right) The Venn diagram represents the number of genes assigned to H3K27ac peaks in GABA and Glu neurons. **b**. Genes assigned to Glu specific peaks are highly expressed in excitatory neurons, while genes assigned to GABA specific peaks are highly expressed in inhibitory neurons. **c**. (Left) An inhibitory neuronal gene, *GAD1*, is engaged in GABA specific peaks and loops. (Right) A glutamatergic neuronal gene, *GRIK4*, is engaged in Glu specific peaks and loops. Gene promoters are highlighted in grey, and their interacting regions are highlighted in blue for Glu and purple for GABA. Boxplots in the right show expression levels of *GAD1* (FDR=2.42e-22) and *GRIK4* (FDR=3.45e-49) in Glu (n=9) and GABA (n=9) cells. TPM: Transcripts Per Kilobase Million. Center, median; box = Q1-Q3; minima, Q1 - 1.5 x IQR; maxima, Q3 + 1.5 x IQR. *FDR<0.05 calculated by DESeq2 (two-sided Wald test). Source data are provided as a Source Data file.

Description of Additional Supplementary Files

File Name: Supplementary Data 1

Description: Coordinates of FIREs and common FIREs in NeuN+ and NeuN− cells as well as GO terms for common FIRE-associated genes.

File Name: Supplementary Data 2

Description: Coordinates of differential- and super-FIREs in NeuN+ and NeuN− cells and their associated genes.

File Name: Supplementary Data 3

Description: Enhancer-promoter interactions in NeuN+ and NeuN− cells.

File Name: Supplementary Data 4

Description: Enhancer-promoter interactions in Glu and GABA neurons.

File Name: Supplementary Data 5

Description: NeuN+ hypoacetylated and NeuN− hyperacetylated genes and their enriched biological processes.

File Name: Supplementary Data 6

Description: AD risk genes and their associated biological pathways.

File Name: Supplementary Data 7

Description: Genes and pathways associated with SCZ and BD GWAS via Glu and GABA H-MAGMA.

Supplementary Reference

1. Seyfried, N. T. *et al.* A Multi-network Approach Identifies Protein-Specific Co-expression in Asymptomatic and Symptomatic Alzheimer's Disease. *Cell Syst.* **4**, 60-72.e4 (2017).

2. Yang, T. *et al.* HiCRep: assessing the reproducibility of Hi-C data using a stratum-adjusted correlation coefficient. *Genome Res.* **27**, 1939–1949 (2017).
