# Supplementary material for: Neuronal and glial 3D chromatin architecture informs the cellular etiology of brain disorders
Source: Nat Commun. 2021 Jun 25;12:3968. doi: 10.1038/s41467-021-24243-0 (PMC8233376; doi:10.1038/s41467-021-24243-0)
Supplement: Supplementary file 2 — Description of Additional Supplementary Files [file 41467_2021_24243_MOESM2_ESM.pdf]

Description of Additional Supplementary Files

File Name: Supplementary Data 1

Description: Coordinates of FIREs and common FIREs in NeuN+ and NeuN− cells as well as GO terms for common FIRE-associated genes.

File Name: Supplementary Data 2

Description: Coordinates of differential- and super-FIREs in NeuN+ and NeuN− cells and their associated genes.

File Name: Supplementary Data 3

Description: Enhancer-promoter interactions in NeuN+ and NeuN− cells.

File Name: Supplementary Data 4

Description: Enhancer-promoter interactions in Glu and GABA neurons.

File Name: Supplementary Data 5

Description: NeuN+ hypoacetylated and NeuN− hyperacetylated genes and their enriched biological processes.

File Name: Supplementary Data 6

Description: AD risk genes and their associated biological pathways.

File Name: Supplementary Data 7

Description: Genes and pathways associated with SCZ and BD GWAS via Glu and GABA H-MAGMA.
