# Supplementary material for: Neuronal and glial 3D chromatin architecture informs the cellular etiology of brain disorders
Source: Nat Commun. 2021 Jun 25;12:3968. doi: 10.1038/s41467-021-24243-0 (PMC8233376; doi:10.1038/s41467-021-24243-0)
Supplement: Supplementary file 10 — Reporting summary [file 41467_2021_24243_MOESM10_ESM.pdf]

nature research

Corresponding author(s): Hyejung Won, Daniel H. Geschwind

Last updated by author(s): Mar 1, 2021

Reporting Summary

Nature Research wishes to improve the reproducibility of the work that we publish. This form provides structure for consistency and transparency in reporting. For further information on Nature Research policies, see our Editorial Policies and the Editorial Policy Checklist.

Statistics

For all statistical analyses, confirm that the following items are present in the figure legend, table legend, main text, or Methods section.

n/a	Confirmed	
[ ]	[x]	The exact sample size (*n*) for each experimental group/condition, given as a discrete number and unit of measurement
[x]	[ ]	A statement on whether measurements were taken from distinct samples or whether the same sample was measured repeatedly
[ ]	[x]	The statistical test(s) used AND whether they are one- or two-sided<br>*Only common tests should be described solely by name; describe more complex techniques in the Methods section.*
[x]	[ ]	A description of all covariates tested
[ ]	[x]	A description of any assumptions or corrections, such as tests of normality and adjustment for multiple comparisons
[ ]	[x]	A full description of the statistical parameters including central tendency (e.g. means) or other basic estimates (e.g. regression coefficient) AND variation (e.g. standard deviation) or associated estimates of uncertainty (e.g. confidence intervals)
[ ]	[x]	For null hypothesis testing, the test statistic (e.g. F, t, r) with confidence intervals, effect sizes, degrees of freedom and P value noted<br>*Give P values as exact values whenever suitable.*
[x]	[ ]	For Bayesian analysis, information on the choice of priors and Markov chain Monte Carlo settings
[x]	[ ]	For hierarchical and complex designs, identification of the appropriate level for tests and full reporting of outcomes
[ ]	[x]	Estimates of effect sizes (e.g. Cohen's *d*, Pearson's *r*), indicating how they were calculated

Our web collection on statistics for biologists contains articles on many of the points above.

Software and code

Policy information about availability of computer code

Data collection	No software or code has been used for data collection.
Data analysis	Custom code used in this study is available through the github repository: https://github.com/thewonlab/NeuN.<br>Software: hiclib (v.0.9), pheatmap (v.1.0.12), HiCRep (v.1.10.0), HiCExplorer (v.2.2.1.1), FIREcaller (v.1.10), FastQC (v.0.11.8), HISAT2 (v.2.1.0), StringTie (v.1.3.5), DESeq2 (v.1.22.2), Cutadapt (v.1.18), Bowtie2 (v.2.3.4.3), Picard (v.2.20.1), MACS2 (v.2.1.0.20150731), DiffBind (v.2.13.1), GimmeMotifs (v0.14.1), gProfileR2 (v.0.1.9), gProfileR (v.0.7.0), H-MAGMA (v.1.08), EWCE (v.1.3.0), LDSC (v1.0.0), Python (v.2.7.0), R (v.3.6.0)

For manuscripts utilizing custom algorithms or software that are central to the research but not yet described in published literature, software must be made available to editors and reviewers. We strongly encourage code deposition in a community repository (e.g. GitHub). See the Nature Research guidelines for submitting code & software for further information.

Data

Policy information about availability of data

All manuscripts must include a data availability statement. This statement should provide the following information, where applicable:

- Accession codes, unique identifiers, or web links for publicly available datasets
- A list of figures that have associated raw data
- A description of any restrictions on data availability

Hi-C data described in this manuscript is available through the PsychENCODE Knowledge Portal with the accession code of syn21760712 [https://www.synapse.org/#!Synapse:syn21760712]. H3K27ac, ATAC-seq, and RNA-seq data from NeuN+ and NeuN− cells are available through syn4566010 [https://www.synapse.org/#!Synapse:syn4566010], GSE83345 [https://www.ncbi.nlm.nih.gov/geo/query/acc.cgi?acc=GSE83345], and syn20545534 [https://www.synapse.org/#!Synapse:syn20545534], respectively. H3K27ac ChIP-seq data from Glu and GABA neurons are available through syn12034263 [https://www.synapse.org/#!Synapse:syn12034263]. Human reference genome and gene definition was obtained from GENCODE [https://www.gencodegenes.org/human/release_19.html] and

UCSC genome browser [http://genome.ucsc.edu].

Field-specific reporting

Please select the one below that is the best fit for your research. If you are not sure, read the appropriate sections before making your selection.

- [x] Life sciences
- [ ] Behavioural & social sciences
- [ ] Ecological, evolutionary & environmental sciences

For a reference copy of the document with all sections, see nature.com/documents/nr-reporting-summary-flat.pdf

Life sciences study design

All studies must disclose on these points even when the disclosure is negative.

Sample size	NeuN+ and NeuN- cells were obtained from the dorsolateral prefrontal cortex (DLPFC) of 4 individuals. No sample size determinations were made. N=4 is a typical sample size for building a Hi-C resource.
Data exclusions	No data was excluded.
Replication	Hi-C data from neurons and glia was generated from 4 biological replicates. We ran HiCRep on each Hi-C dataset to demonstrate (1) samples cluster based on cell types (Neurons vs. Glia) rather than individuals, and (2) high reproducibility among neuronal/glia Hi-C datasets (Stratum-adjusted correlation coefficient ~0.9). We also overlapped Hi-C result with other functional genomic datasets to further validate our findings.
Randomization	No randomization was conducted. Since NeuN+ (or NeuN-) Hi-C libraries displayed strong reproducibility, we merged all NeuN+ (or NeuN-) Hi-C data together to generate a combined high-resolution contact matrix. Moreover, NeuN+/NeuN- libraries were built from the same individual and all samples and datasets have been included in our analysis, so there was no need for randomization.
Blinding	Investigators were not blinded. Both NeuN+ and NeuN- data were treated equally with the same computational analytic pipeline.

Reporting for specific materials, systems and methods

We require information from authors about some types of materials, experimental systems and methods used in many studies. Here, indicate whether each material, system or method listed is relevant to your study. If you are not sure if a list item applies to your research, read the appropriate section before selecting a response.

Materials & experimental systems

n/a	Involved in the study	
[ ]	[x]	Antibodies
[x]	[ ]	Eukaryotic cell lines
[x]	[ ]	Palaeontology and archaeology
[x]	[ ]	Animals and other organisms
[ ]	[x]	Human research participants
[x]	[ ]	Clinical data
[x]	[ ]	Dual use research of concern

Methods

n/a	Involved in the study	
[x]	[ ]	ChIP-seq
[ ]	[x]	Flow cytometry
[x]	[ ]	MRI-based neuroimaging

Antibodies

Antibodies used	anti-NeuN-488, Millipore, Cat# MAB377X
Validation	This antibody has been previously used to sort neuronal nuclei from the brain homogenate. Validation and characterization of this antibody can be found in these references: Gorkin, D. U. et al. Common DNA sequence variation influences 3-dimensional conformation of the human genome. Genome Biol. 20, 255 (2019); Kundakovic, M. et al. Practical Guidelines for High-Resolution Epigenomic Profiling of Nucleosomal Histones in Postmortem Human Brain Tissue. Biol. Psychiatry 81, 162–170 (2017).

Human research participants

Policy information about studies involving human research participants

Population characteristics	Detailed sample information is available in Supplementary Table 1.
Recruitment	We have used postmortem brain specimens, so there was no recruitment involved.
Ethics oversight	Prefrontal cortex with no history of neurological disease was procured from tissue collections at the Department of Neuroscience at Yale University School of Medicine. Additional specimens were procured from the Brain and Tissue Bank at the University of Maryland. Tissue was collected after obtaining next of kin consent and with approval by the institutional review boards at the Yale University School of Medicine, the National Institutes of Health, and at each institution from which tissue specimens were obtained. Tissue specimens were handled in accordance with ethical guidelines and regulations for the research use of human brain tissue set forth by the NIH and the WMA Declaration of Helsinki.

Note that full information on the approval of the study protocol must also be provided in the manuscript.

Flow Cytometry

Plots

Confirm that:

- [x] The axis labels state the marker and fluorochrome used (e.g. CD4-FITC).
- [x] The axis scales are clearly visible. Include numbers along axes only for bottom left plot of group (a 'group' is an analysis of identical markers).
- [x] All plots are contour plots with outliers or pseudocolor plots.
- [x] A numerical value for number of cells or percentage (with statistics) is provided.

Methodology

Sample preparation	Approximately 300 mg of cortical gray matter is dissected from fresh frozen postmortem human brain samples, specifically derived from the dorsolateral prefrontal cortex. The tissue is then homogenized and undergoes sucrose gradient ultracentrifugation for nuclei extraction. At this stage, the sample is incubated with Neuronal Nuclei NeuN-Alexa-488 conjugated antibody (Millipore, Cat# MAB377X) in order to differentiate NeuN+ (neuronal) from NeuN- (non-neuronal) nuclei in fluorescence-activated cell sorting. The sample is then stained with DAPI (Thermoscientific, Cat# 62248) to preferentially sort nuclei from cells not undergoing cell division.
Instrument	BD™ FACSAria II (SOP) Cell Sorter, enclosed in a Baker BioPROtect® III Hood
Software	BD FACSDivaTM software
Cell population abundance	Roughly 0.6 to 0.7 million of each NeuN+ and NeuN- nuclei are recovered per 100 mg of dissected gray matter with a NeuN +:NeuN- ratio of approximately 1:1. Gentle sort settings with a 100 µm nozzle and sheath fluid pressure at 20 psi were used to preserve structural integrity of nuclei and maintain reasonable collection volumes. Samples of sorted NeuN+ and NeuN- nuclei were then visually inspected under a microscope to confirm nuclei extraction/purification and as a form of quality control.
Gating strategy	Preliminary forward scatter and side scatter gates were positioned to include only single, viable nuclei in the analysis by excluding events with low side scatter and high forward scatter, typical of debris and clumps of nuclei. The gating scheme for DAPI was set to be inclusive of only DAPI-stained nuclei. Our NeuN gating criteria then operated under a strategy of maximizing specificity over sensitivity to ensure that NeuN+ and NeuN- nuclei could be confidently sorted into respective collections without additional debris. FITC gating for Alexa-488 fluorescence was used to discriminate NeuN+ from NeuN-.

- [x] Tick this box to confirm that a figure exemplifying the gating strategy is provided in the Supplementary Information.
